# Obesity and Bariatric Surgery in Australia: Future Projection of Supply and Demand, and Costs

**DOI:** 10.1007/s11695-022-06188-5

**Published:** 2022-07-08

**Authors:** Sithara Wanni Arachchige Dona, Mary Rose Angeles, Dieu Nguyen, Lan Gao, Martin Hensher

**Affiliations:** 1 Deakin Health Economics, School of Health and Social Development, Deakin University, Locked Bag 20000, Geelong, VIC 3220 Australia; 2 Menzies Institute for Medical Research, University of Tasmania, Medical Sciences Precinct, 17 Liverpool Street, Hobart, TAS 7000 Australia

**Keywords:** Bariatric surgery, Eligibility, Supply and demand, Costs

## Abstract

**Introduction:**

The prevalence of obesity is increasing in developed countries, including Australia. There is evidence that bariatric surgery is effective in losing weight and reducing risk of chronic diseases. However, access to bariatric surgery remains limited in the public health sector.

**Method:**

We modelled population-based estimates of the likely numbers of people eligible for bariatric surgery in Australia using the recent Australian New Zealand Metabolic and Obesity Surgery Society (ANZMOSS) framework and estimated the potential costs that would be incurred from primary and subsequent reoperations in both public and private sector.

**Results:**

The annual number of newly eligible patients is expected to rise, and hence the gap in demand is increasing relative to current baseline supply. If a 5-year program to treat all currently eligible patients was implemented, the maximum yearly demand is projected to be 341,343 primary surgeries, more than eight times the existing capacity of public and private sector, which can only offer 41,534 surgeries/year. A nine-fold increase is expected if we treat currently eligible patients over a 5-year program and all newly eligible patients as they occur each year.

**Conclusion:**

Our results highlighted the currently highly skewed distribution of bariatric surgeries between the private and public sectors. Improving access would bring substantial benefits to many Australians, given the demonstrated cost-effectiveness and cost savings. This requires a major increase in resourcing for publicly-funded access to bariatric surgery in the first instance. A national review of priorities and resourcing for all modes of obesity treatment is required in Australia.

**Graphical abstract:**

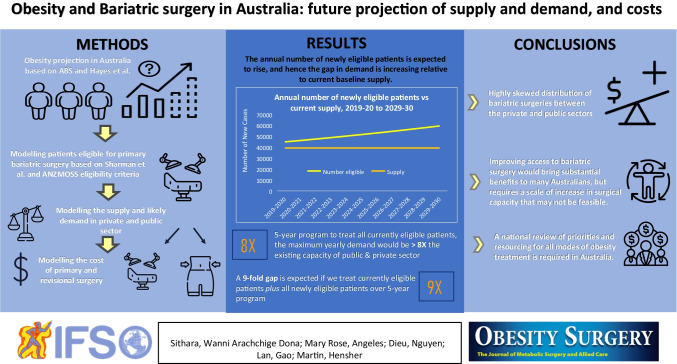

**Supplementary Information:**

The online version contains supplementary material available at 10.1007/s11695-022-06188-5.

## Introduction

The prevalence of obesity has been increasing in most high-income countries, including Australia. The most recent 2017–2018 National Health Survey found that 67% of adults were affected by either overweight or obesity in Australia [1]. This was an increase from 63.4% in the 2014–2015 survey, and this change was driven by the increase in the number of adults with obesity rather than those affected by overweight [1].

Obesity has become a major concern especially because it is a major risk factor for chronic conditions, including but not limited to type II diabetes mellitus (T2DM), cardiovascular disease, and musculoskeletal disorders (e.g. arthritis) [2]. In its most recent Burden of Disease Study, the Australian Institute of Health and Welfare noted that overweight and obesity was the second largest cause of total disease burden, responsible for 8.4% of total DALY burden in Australia in 2018 [3]. There are a range of strategies available both to prevent obesity and to treat it following onset. Bariatric surgery is an important treatment option for obesity in Australia and overseas.

There is evidence that bariatric surgery is effective in losing weight and reducing risk of chronic diseases such as cardiovascular diseases, non-alcoholic steatohepatitis, and diabetes [4–6]. Bariatric surgery has been found to be cost-effective in people with obesity (i.e. BMI > 35) [7] compared to non-surgical treatments, and cost-saving for people with higher initial body mass index, people with T2DM, and younger cohort [7, 8]. In particular, laparoscopic Roux-en-Y gastric bypass (LRYGB) is more cost-effective than no treatment or medical management [9], and superior to laparoscopic sleeve gastrectomy (LSG), laparoscopic adjustable gastric banding (LAGB) [4].

Although the prevalence of obesity is increasing and the bariatric surgical pathway has been shown to be both clinically effective and cost effective, the availability of publicly funded bariatric surgery remains limited in Australia [8]. Sharman et al. in 2018 estimated the potentially eligible Australian population for bariatric surgery, based on the 2011–2013 Australian Health Survey and the recommendations for the eligibility made by the National Health and Medical Research Council guidelines for the management of overweight and obesity [10]. They concluded that the capacity for bariatric surgery was not sufficient to meet demand even if only 5% of eligible Australian adults sought surgery.

In 2020, the Australian New Zealand Metabolic and Obesity Surgery Society (ANZMOSS) developed a National Framework for public bariatric surgery, which sought to provide recommendations to assist Australian public health authorities in developing effective and sustainable surgical care to the most appropriate populations [11]. The ANZMOSS Framework incorporated the Edmonton Obesity Scoring System (EOSS) as a key element of its recommendations on eligibility criteria for public bariatric surgery [11]. The ANZMOSS Framework was complementary to the first Australian National Framework for clinical obesity services, but neither has been formally adopted by Australian health departments or health system managers [12].

We modelled population-based estimates of the likely numbers of people eligible for bariatric surgery in Australia using the ANZMOSS Framework and estimated the potential costs that would be incurred from primary procedures and subsequent reoperations. Our aim was to simulate the likely resource impacts and feasibility of applying the ANZMOSS Framework to the eligible Australian population.

## Method

Future predictions of population eligibility and demand estimates for bariatric surgery for each year until 2029–2030 were calculated as follows and Appendix 1.1 elaborates the methodology in depth.

1. Estimating the population with obesity in Australia based on the Australian Bureau of Statistics (ABS) 2017–2018 National Health Survey and the projections of Hayes et al. [13].
2. Estimating the population eligible for primary bariatric surgery based on Sharman et al.’s eligibility estimates [10] and the ANZMOSS eligibility criteria [11]. The estimated eligible population based on the ANZMOSS eligibility criteria incorporated the EOSS classification (Table 1), which was not used by Sharman and the NHMRC. Based on the above eligibility, we modelled patients with poorly controlled T2DM with medication in obesity class I, people with established obesity-related chronic disease (hypertension, type 2 diabetes, sleep apnoea, osteoarthritis), or established end-organ damage (myocardial infarction, heart failure, stroke in obesity class II and III, including people with subclinical risk factors in obesity class III (Table 1).
3. Estimating patients becoming newly eligible for primary bariatric surgery.
4. Estimating likely uptake of bariatric surgery.

**Table 1:** Eligibility criteria of ANZMOSS recommendations with EOSS classification

BMI/obesity class	Age	ANZMOSS EOSS eligibility and/or additional	National Health and Medical Research Council recommendation*
BMI > 35**–**40 (obese class II)	18–65-year-old	EOSS 2 and 3 Additionally •Documented previous weight loss attempts •Absence of contraindications •Smoking should be stopped prior to BS	Recommended for those with resistant Class 2 obesity (BMI 35–39.9 kg/m2) and obesity related comorbidities
BMI > 40 (obese class III)	18–65	EOSS 1–3 Additionally •Documented previous weight loss attempts •Absence of contraindications •Smoking should be stopped prior to BS ** 18–65 yrs, BMI > 40 and EOSS 4: require a skilled bariatric team	Resistant class 3 obesity (BMI > 40 kg/m2)
BMI: > 40 (obese class III)	65–70-year-old	EOSS: 2–3 Additionally •Documented previous weight loss attempts •Absence of contraindications •Smoking should be stopped prior to BS	
BMI > 30**–**35 (obese class I)	-	EOSS not applicable T2DM for < 10 years or has favourable C-peptide level which is poorly controlled with medication	For consideration for adults with resistant class 1 obesity and (BMI 30–34.9 kg/m2) and poorly controlled T2DM and are at increased cardiovascular risk
BMI > 35	-	With established diabetes	

EOSS 1: presence of obesity-related subclinical risk factors (ex: borderline HTN, impaired fasting glucose levels, elevated levels of liver enzymes), mild physical symptoms (ex: dyspnoea on moderate exertion, occasional aches and pains, fatigue), mild psychopathology, mild functional limitations and/or mild impairment of wellbeing. EOSS 2: presence of established obesity-related chronic disease (hypertension, type 2 diabetes, sleep apnoea, osteoarthritis), moderate limitation in activities of daily living and or well-being. EOSS 3: established end-organ damage ex: MI, heart failure, stroke, significant psychopathology, significant functional limitations and or impairment of well-being. EOSS 4: end stage disease that will require a clinical assessment to determine whether it is palliative before exclusion from surgery. *Source: ANZMOSS & Collaborative Public Bariatric Surgery Taskforce.* *According to the National Health and Medical Research Council guideline for the Management of Overweight and Obesity in Primary Care, Bariatric surgery might be considered for adults with BMI > 40 kg/m2, or adults with BMI > 35 kg/m2 and comorbidities that may improve with weight loss, taking into account the individual situation or people with a BMI > 30 kg/m2 who have poorly controlled type 2 diabetes and are at increased cardiovascular risk. Source: [10, 14]

### Description of Supply and Demand Model

#### Bariatric Surgical Capacity

The historical numbers of total bariatric surgery procedures were obtained from AIHW. Current capacity was based on the uptake rates of bariatric surgery, the proportion of the population with private health insurance, and the number of procedures in the private and public sectors (see Appendix 1.2 for in-depth information).

Current supply and potential future demand (using the ANZMOSS recommendations based on corresponding EOSS classification) were then compared for three different provision scenarios (Appendix 2).

Scenario 1: Newly eligible cases only.

Scenario 2: Existing cases only — assuming a 5-year program to treat all existing eligible cases (as of 2019–2020).

Scenario 3: Treating all newly eligible cases and a 5-year program to treat existing patients.

See Appendix 2 for the description of the scenarios.

### Costs of Primary and Revisional Surgeries

#### Model Structure

We developed a dynamic decision tree model based on the Western Australia bariatric reoperation admission matrix from 2007 to 2016 to estimate the likely number of revisions arising from the primary bariatric surgery, which were used to estimate the total costs of bariatric surgeries. The model structure consists of two health states: patients with or without adverse events requiring subsequent reoperations. See Appendix 3 for further information including data inputs.

## Results

**Fig. 1:**
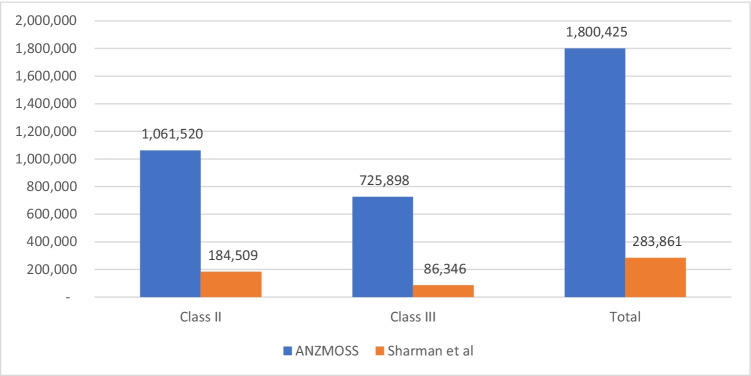
Adults aged 18–70 potentially eligible for primary bariatric surgery, 2021–2022

Estimates of the population potentially eligible for primary bariatric surgery were calculated based on the estimated population of 4,919,600 adults with obesity and aged 18–70 obtained from the 2017–2018 National Health Survey [15]. Of those, 1,800,425 (34.6%) were estimated to be potentially eligible for primary bariatric surgery by 2021–2022 based on the ANZMOSS eligibility criteria. The estimated number of eligible people was higher in obesity class II compared to class III, and the ANZMOSS criteria generated a much higher estimate of eligible patients than Sharman’s original method (Fig. 1).

### Demand Scenarios: Estimates of Demand for Primary Procedures

**Fig. 2:**
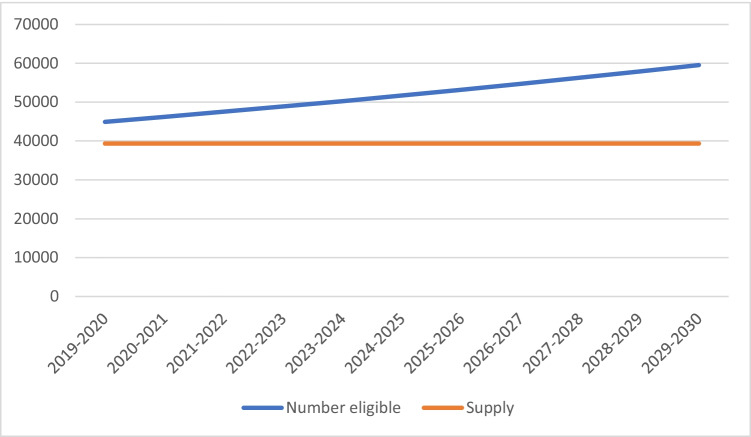
Estimated annual number of newly eligible patients versus current supply, 2019–2020 to 2029–2030

Under scenario 1, the annual number of people newly eligible for primary surgery increases over time as shown in Fig. 2. The annual demand from newly eligible persons in 2019–2020 was estimated to be 44,921, which was expected to grow to 59,551 by 2029–2030, compared with baseline (2018–2019) supply of 41,534 procedures.

**Fig. 3:**
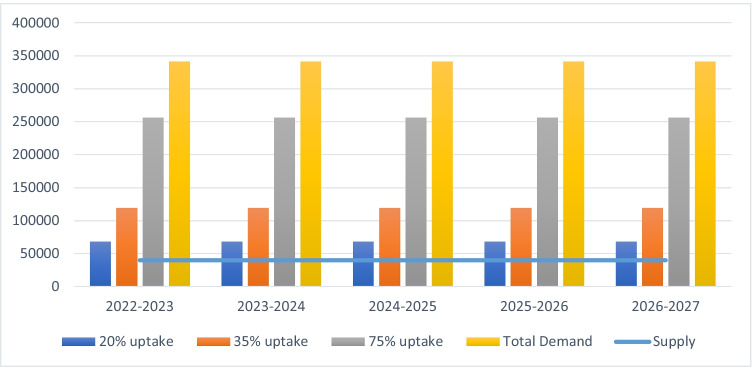
Treating existing patients for primary procedures only assuming a 5-year program

Figure 3 shows the results of model scenario 2, representing a 5-year program to treat existing patients (using 2019–2020 as the baseline year) between 2022–2023 and 2026–2027. The maximum yearly demand (i.e. all eligible patients) is projected to be 341,343 primary surgeries, more than eight times the existing capacity of the healthcare system, which can only offer 41,534 surgeries per year. Even assuming only a 20% uptake rate, total demand would still be higher than the available number of surgical procedures.

**Fig. 4:**
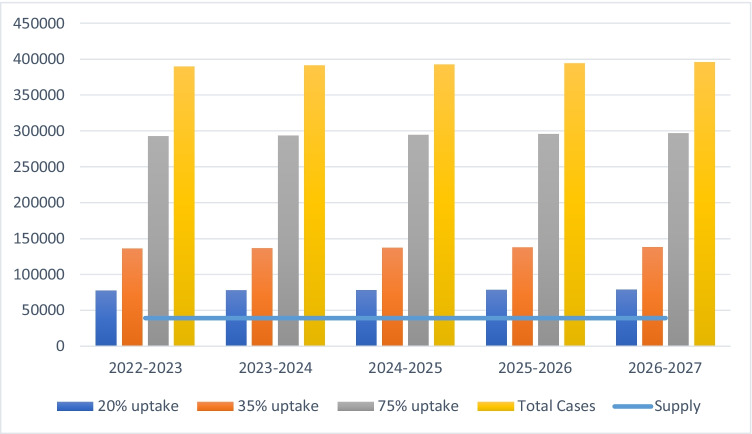
Treating newly eligible patients and existing eligible patients for primary procedures in a 5-year program

In scenario 3, if we treat existing patients as of 2019–2020 over the 5-year program *and* all newly eligible cases as they occur each year, Fig. 4 shows the estimated level of demand versus the currently available number of surgeries. Scenario 3 obviously entails an even larger (nine-fold) excess of demand over current supply.

**Fig. 5:**
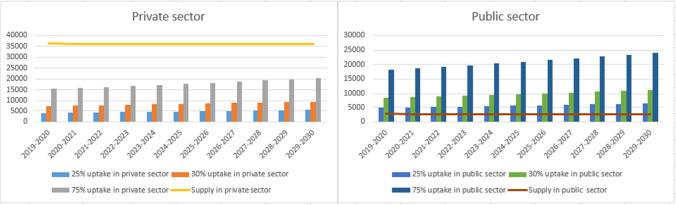
Supply and demand in private and public sector for scenario 1 (newly eligible patients)

Demand and supply currently differ significantly between the public and the private sectors. For example, the national total of 41,534 of bariatric procedures in 2018–2029 comprised 38,512 procedures performed in the private sector, and only 3022 from the public sector. Some 46% of the Australian population have private health insurance hospital cover. While some uninsured patients do pay out-of-pocket for bariatric surgery in the private sector, the vast majority of the 54% of uninsured Australians could be expected to rely on public hospital services only. Figure 5 illustrates the relationship between supply and demand in both sectors for newly eligible patients (scenario 1). Currently available private sector capacity is more than sufficient to cover newly eligible patients each year (scenario 1); but current public sector capacity cannot meet even 20% of newly eligible patients in the population without private health insurance. Scenarios 2 and 3 further exceed public sector capacity (Appendix 2). Under scenarios 2 and 3, the private sector could currently meet 20% of the existing eligible patients plus newly eligible patients, but private capacity is not sufficient to meet 35% or 75% uptake. The disparity between current capacity and potential demand remains much larger in the public sector than the private.

### Potential Demand for Revisional Surgery

Table 2 shows the likely stream of future revisions over a 10-year period, given the primary procedures under each scenario and uptake level per year (full details are noted in Appendix 4–7). For example, under scenario 1, 20% uptake, 9773 primary procedures would occur in 2022–2023, generating a stream of 637 revisions that would occur over the following 10 years. For scenario 2, 68,269 primary surgeries were estimated per year from 2022 to 2026 for an uptake level of 20% generating an estimated 4448 revision surgeries over the following 10 years. Similarly, under scenario 3 with 35% uptake, in 2025–2026, 138,082 primary procedures would generate a future stream of 8997 revisions over the following 10 years. Unsurprisingly, these revisions were mostly driven by LAGB, which required reoperations in 32.35% of cases as opposed to only 2.4% of RYGB (2.4%) and 0.87% SG (Appendix 4–6).

**Table 2:** Potential future stream of demand for revision from primary procedures

Uptake level	2022–2023	2023–2024	2024–2025	2025–2026	2026–2027	2027–2028	2028–2029	2029–2030
Scenario 1: newly eligible patients only								
20%	637	655	674	693	713	733	754	776
35%	1,114	1,146	1,179	1,213	1,247	1,283	1,320	1,358
75%	2,388	2,456	2,526	2,599	2,673	2,750	2,829	2,910
Scenario 2: existing patients only — assumes a 5-year program to treat all existing (2019**–**2020) eligible patients								
20%	4,448	4,448	4,448	4,448	4,448	-	-	-
35%	7,785	7,785	7,785	7,785	7,785	-	-	-
75%	16,681	16,681	16,681	16,681	16,681	-	-	-
Scenario 3: treating new eligible patients and a 5-year program to treat existing eligible patients (as of 2019**–**2020)								
20%	5,085	5,103	5,122	5,141	5,161	733	754	776
35%	8,899	8,931	8,964	8,997	9,032	1,283	1,320	1,358
75%	19,069	19,138	19,208	19,280	19,354	2,750	2,829	2,910

“-” not applicable

### Total Costs

Table 3 presents the cost of bariatric surgery from different scenarios including the cost for the public, private, and the combined sectors. The estimated cost of current (2018–2019) activity was AUD $486 million. Under scenario 1, the total cost of the program is less than or close to current total costs even at 75% uptake. However, even at the lowest (20%) uptake level, public sector costs would be close to double their baseline levels, indicating a need for significant resource reallocation to the public sector even under this most conservative scenario.

**Table 3:** Total costs in millions

Uptake level	Split	2018–2019	2022–2023	2023–2024	2024–2025	2025–2026	2026–2027	2027–2028	2028–2029	2029–2030
Baseline	Public	35.4	-	-	-	-	-	-	-	-
	Private	450.5	-	-	-	-	-	-	-	-
	Total	485.9	-	-	-	-	-	-	-	-
Scenario 1: newly eligible patients only										
20%	Public	-	65.2	67.5	69.9	72.3	74.8	77.4	80.1	82.8
	Private	-	55.5	57.5	59.5	61.6	63.7	65.9	68.2	70.5
	Total	-	120.8	125.0	129.4	133.9	138.5	143.3	148.3	153.3
35%	Public	-	114.1	118.1	122.3	126.5	130.9	135.4	140.1	144.9
	Private	-	97.2	100.6	104.2	107.8	111.5	115.4	119.3	123.4
	Total	-	211.3	218.8	226.4	234.3	242.5	250.8	259.4	268.3
75%	Public	-	244.5	253.1	262.0	271.1	280.6	290.2	300.2	310.5
	Private	-	208.3	215.6	223.2	231.0	239.0	247.2	255.7	264.5
	Total	-	452.8	468.8	485.2	502.1	519.5	537.5	555.9	575.0
Scenario 2: existing patients only — assumes a 5-year program to treat all existing (2019**–**2020) eligible patients										
20%	Public	-	455.5	458.5	461.6	464.6	467.7	15.2	15.2	15.2
	Private	-	388.0	390.6	393.2	395.8	398.4	13.0	13.0	13.0
	Total	-	843.5	849.1	854.8	860.4	866.1	28.2	28.2	28.2
35%	Public	-	797.1	802.4	807.8	813.1	818.4	26.7	26.7	26.7
	Private	-	679.0	683.6	688.1	692.6	697.2	22.7	22.7	22.7
	Total	-	1,476.1	1,486.0	1,495.9	1,505.8	1,515.6	49.4	49.4	49.4
75%	Public	-	1,708.1	1,719.5	1,731.0	1,742.4	1,753.8	57.1	57.1	57.1
	Private	-	1,455.0	1,464.8	1,474.5	1,484.2	1,494.0	48.7	48.7	48.7
	Total	-	3,163.1	3,184.3	3,205.5	3,226.6	3,247.8	105.8	105.8	105.8
Scenario 3: treating new eligible patients and a 5-year program to treat existing eligible patients (as of 2019–2020)										
20%	Public	-	520.7	526.0	531.5	536.9	542.5	92.6	95.3	98.0
	Private	-	443.6	448.1	452.7	457.4	462.1	78.9	81.2	83.5
	Total	-	964.3	974.2	984.2	994.3	1004.6	171.5	176.5	181.5
35%	Public	-	911.2	920.6	930.1	939.6	949.4	162.1	166.8	171.6
	Private	-	776.2	784.2	792.3	800.4	808.7	138.1	142.1	146.1
	Total	-	1,687.5	1,704.8	1,722.3	1,740.1	1,758.1	300.2	308.8	317.7
75%	Public	-	1,952.6	1,972.7	1,993.0	2,013.5	2,034.4	347.4	357.3	367.6
	Private	-	1,663.4	1,680.4	1,697.7	1,715.2	1,733.0	295.9	304.4	313.2
	Total	-	3,616.0	3,653.1	3,690.7	3,728.8	3,767.3	643.3	661.8	680.8

“-” not applicable

Under scenarios 2 and 3 (larger surgical programs), costs would be significantly higher than currently in both public and private sectors at all uptake levels. In all scenarios, though, the increase in costs is proportionately greater for the public sector than the private.

In addition, under scenario 3, revisional surgery arising from the primary surgery scenarios would cost up to $34 million (20%), $60 million (35%), and $129 million (75%) over the 10-year period, some of which would be transferred to the public health system. See Appendix 8 for costs for each surgery types. Only direct medical costs were included in the costs model due to availability issues.

## Discussion

Our study found that demand and supply of primary bariatric surgery currently differ significantly between the public and the private sectors. Existing capacity in both sectors is unlikely to be able to offer surgery to more than a fraction of all patients who might currently be eligible for surgery under the ANZMOSS guidelines. Any attempt to increase access to bariatric surgery in Australia requires significant additional resources to be allocated to the public hospital system in the first instance. As the number of people with obesity increases, clear eligibility criteria for bariatric surgery and the capacity to meet that demand remain unresolved issues in the Australian health system. Where earlier NHMRC guidelines primarily used BMI as the main eligibility criterion [14], the ANZMOSS guidelines have sought to introduce EOSS as an eligibility criterion along with BMI, to allow more precise targeting of primary bariatric surgery. However, the introduction of EOSS as eligibility criteria appears to lead to higher numbers of potentially eligible patients than did Sharman’s modelling of the earlier NHMRC guidelines.

ANZMOSS are not alone in recommending the use of EOSS to guide eligibility for bariatric surgery [11, 16]. Yet one of the challenges in Australia is that robust estimates of the prevalence and distribution of EOSS in the Australian population are not readily available. Most of the available studies reported the proportions of EOSSs within populations but not separated by obesity classes [17–19]. To our knowledge, only one Australian study has reported EOSS along with BMI [16]; however, EOSS scores for BMI above 35 were not reported. This is important as EOSS is not considered an eligibility criterion for bariatric surgery in obesity class one, but only in the other two classes of obesity under the ANZMOSS criteria. In the absence of Australian data, our model has employed calibration data from the most applicable international evidence on the proportions of EOSS within each obesity class. Of note, the chosen USA study was relatively old; therefore, the results of our analysis might change when newer and better evidence becomes available in the future.

While the growth rate of the population with obesity is expected to increase [13], the EOSS stages are also reported to advance over time. For example, Canning et al. suggest that those in EOSS stages 1 and 2 may transition into higher EOSS stage over time [20], thus becoming eligible. However, relevant data was not available to capture changes in the EOSS stages and obesity classes over time, and therefore it is not reflected in our model.

Moreover, there is evidence in the literature that EOSS stages 3 and 4 are more prone to complications than EOSS stages 0 to 2 [16]. However, we did not consider this difference of effect in our model as eligibility criteria were based on ANZMOSS where EOSS stages for each obesity class were reported. An additional criterion for ANZMOSS was the clinical assessment of eligibility, such as contraindications and previous attempts to lose weight, but no data were available to incorporate clinical assessment into this model.

Our study only considered a single treatment modality for patients affected by obesity — bariatric surgery. Previous evidence has tended to show bariatric surgery as being significantly more effective than pharmacotherapy in isolation, but a number of authors have recently suggested that more promising pharmacotherapies for obesity may soon be available [21, 22]. New drugs or more effective ways of optimising multimodal treatments (potentially combining bariatric surgery, drug therapy and other interventions)) may offer more promising outcomes in future and should be a priority for research and evaluation. Equally, given the increasing acceptance that the ultimate goal of obesity management is to prevent chronic diseases [23], the introduction of more effective pharmacotherapies would, in the long term, provide a more feasible and sustainable means of addressing obesity at large scale than could an expanded obesity surgery program. Policy makers will need to ensure they have strong and up-to-date evidence on the relative effectiveness of new and combined treatments over coming years to support wise decision making in this area.

Under the ANZMOSS guideline, failures in previous weight loss attempts, resistant obesity, and T2DM that medications could not control are considered additional priority or eligibility criteria for bariatric surgery (Table 1). These factors were modelled in our study, and our findings indicated a relatively substantial demand for bariatric surgery. However, due to limited available data, we were not able to model patients with hypertension, sleep apnoea, well-controlled diabetes, and non-alcoholic fatty liver disease (NAFLD) in isolation. Data limitations inevitably make it hard to model comprehensively all the individual comorbidities and risk factors at a population level, even though the use of EOSS within the ANZMOSS guidelines could support more fine-grained clinical prioritisation decisions at the level of individual patients.

Our results highlight the currently highly skewed distribution of bariatric surgery activity (and hence access to surgery) between the private and public sectors. Current public sector bariatric surgery capacity in Australia can cover only a small fraction of likely population needs, even under the most conservative of the scenarios we examined. There are plausible arguments that improving access to bariatric surgery would bring substantial benefits to many Australians, given the demonstrated cost-effectiveness of bariatric procedures. According to a systematic review and economic evaluation of LAGB for patients with mild to moderate obesity, the ICER of LAGB compared to a non-surgical comparator was £20,159 at 2 years, £4858 at 5 years, and £1634 at 20 years [7]. Another study investigating the cost-effectiveness of four bariatric surgeries compared to standard non-surgical management for all patients affected by obesity with BMI > 30 reported that RYGB had a QALY gain of 0.5 with an incremental cost of $20,000, leading to an ICER of $37,423. SG and LAGB were noted as less costly but less effective than RYGB, while BPD/DS is more expensive and more effective [24]. Meanwhile, the ICER of SG, LAGB, and BPD compared to standard care ranged from $29,000 to $47,000 per QALY [24]. Doing so would require a major increase in resourcing for publicly funded access to bariatric surgery in the first instance. Given the scale of the gap between available public capacity and potential need (and, indeed, the private sector gap in scenarios 2 and 3), the real constraint to expanding access is likely to be the availability of appropriately qualified surgeons, rather than financing per se. Our results show that the flow of newly eligible cases could potentially be met within or close to existing surgical supply (albeit with the need to redistribute resources between privately and publicly funded care). Yet they also show that attempting to treat any significant proportion of the stock of the 1.8 million people potentially already eligible for bariatric surgery under the ANZMOSS criteria would require ramping up capacity by a factor of eight to nine times current levels. Would it ever be feasible, desirable, or even reasonable to invest in training the number of additional surgeons required to deal with this existing eligible population, many of whom would then be out of work after a few years, once this “backlog” had been cleared? Alternatively, is there scope for greatly improving the productivity of bariatric surgeons and hospital services to treat many more patients via radically redesigned care models, for example by emulating the ultra-low-cost Narayana Health model from India, which incorporated “innovative technology and a highly efficient delivery system” to increase surgeon capacity in a high-volume, low-cost system [25]? The Australian Government should support research on novel medications and multimodal treatments for obesity and actively seek to understand the implications of emerging trends in obesity management [21, 26].

## Conclusion

The ANZMOSS Framework is to be applauded and welcomed in its aim of improving decision-making on eligibility for bariatric surgery. Our results indicate, however, that in the absence of finer-grained Australian data on the actual distribution of EOSS scores across different obesity classes in Australia, the ANZMOSS criteria might inadvertently render more people eligible than earlier NHMRC guidelines. If EOSS scores are to be used to guide decision-making, there is an urgent need for rigorous local research to establish the true prevalence and distribution of EOSS scores by obesity class, and also to explore patient perceptions of their likelihood of taking up bariatric surgery were it available to them, to allow more accurate estimates of both eligibility and likely uptake.

Our results further highlight the disparity in funding and capacity for bariatric surgery between the private and public hospital sectors in Australia. Even if policy makers sought only to run a modest bariatric surgery program, sufficient to deal only with newly eligible patients as they emerged, a substantial increase in funding (and reallocation of the relevant surgical workforce) towards public hospitals would still be required. Equally, our results indicate that very large numbers of Australians could potentially benefit from bariatric surgery, yet currently have next to no chance of accessing this service if they wanted it. The potential scale of the challenge highlighted by our results suggests the need for a deeper and wider debate on the aims, methods, and costs of all potential delivery models required to treat and manage obesity in Australia, not just bariatric surgery. National strategy on obesity in Australia has overwhelmingly focused on prevention. It is time for a major national review of all effective management and treatment options for the millions of Australians who are already affected by obesity. This review would support the development of an integrated national strategy to fund and deliver high quality, cost-effective interventions across nutrition, physical activity, and both medical and surgical treatments, allowing the selection of an optimal mix of different, mutually supporting treatment and management modes.

## Supplementary Information

Below is the link to the electronic supplementary material.

Supplementary file1 (DOCX 256 KB)
