# Supplementary material for: Obesity and Bariatric Surgery in Australia: Future Projection of Supply and Demand, and Costs
Source: Obes Surg. 2022 Jul 8;32(9):3013–22. doi: 10.1007/s11695-022-06188-5 (PMC9392713; doi:10.1007/s11695-022-06188-5)
Supplement: Supplementary file 1 — Supplementary file1 (DOCX 256 KB) [file 11695_2022_6188_MOESM1_ESM.docx]

APPENDICES

Table of Contents

[Appendix 1: Methodology and Data inputs 1](#_Toc106359274)

[Appendix 1.1: Methodology in detail for the future prediction of population estimates for bariatric surgery: 1](#_Toc106359275)

[Appendix 1.2: Data inputs and parameters 3](#_Toc106359276)

[Appendix 1.3: Data inputs and parameters for Cost and revisional surgeries 4](#_Toc106359277)

[Appendix 1.4: Data inputs and parameters for Cost Analysis 6](#_Toc106359278)

[Appendix 2 Capacity model (Combined Private and Public) 9](#_Toc106359279)

[Appendix 2.1 Bariatric surgical capacity and Description of Scenarios 9](#_Toc106359280)

[Appendix 2.2 Capacity model 11](#_Toc106359281)

[Appendix 3: Decision tree 14](#_Toc106359282)

[Appendix 4: Primary and Potential revisions arising from the primary surgery 16](#_Toc106359283)

[Appendix 4.1 Total for Newly eligible patients only (Combined figures of private and public sector) 16](#_Toc106359284)

[Appendix 4.2 Total Existing patients Only - assumes a 5-year program to treat all existing (2019-20) eligible patients 17](#_Toc106359285)

[Appendix 4.3 total Newly Eligible patients plus a 5-year program to treat existing eligible patients (as of 2019-20) 18](#_Toc106359286)

[Appendix 5: Primary and Potential revisions arising from the primary surgery in the Private sector 19](#_Toc106359287)

[Appendix 5.1 Newly eligible patients only 19](#_Toc106359288)

[Appendix 5.2 Existing patients Only - assumes a 5-year program to treat all existing eligible patients (2019-20) 20](#_Toc106359289)

[Appendix 5.3 Treating New Eligible patients plus a 5-year program to treat existing eligible patients (as of 2019-20) 22](#_Toc106359290)

[Appendix 6: Primary and Potential revisions arising from the primary surgery in the public sector 23](#_Toc106359291)

[Appendix 6.1 Newly eligible patients only 23](#_Toc106359292)

[Appendix 6.2 Existing patients Only - assumes a 5-year program to treat all existing (2019-20) eligible patients 24](#_Toc106359293)

[Appendix 6.3 Treating New Eligible patients and a 5-year program to treat existing eligible patients (as of 2019-20) 25](#_Toc106359294)

[Appendix 7: Distribution of Revisions Over 10-year time horizon 27](#_Toc106359295)

[Appendix 7.1 Total (private plus private sector) 27](#_Toc106359296)

[Appendix 7.1.1 Newly eligible patients only 27](#_Toc106359297)

[Appendix 7.1.2 Existing patients Only - assumes a 5-year program to treat all existing (2019-20) eligible patients 28](#_Toc106359298)

[Appendix 7.1.3 Treating New Eligible patients and a 5-year program to treat existing eligible patients (as of 2019-20) 30](#_Toc106359299)

[Appendix 7.2 Total (private sector) 31](#_Toc106359300)

[Appendix 7.2.1 Newly eligible patients only 31](#_Toc106359301)

[Appendix 7.2.2 Existing patients Only - assumes a 5-year program to treat all existing (2019-20) eligible patients 32](#_Toc106359302)

[Appendix 7.2.3 Existing patients Only - assumes a 5-year program to treat all existing (2019-20) eligible patients 33](#_Toc106359303)

[Appendix 7.3 Total (public sector) 34](#_Toc106359304)

[Appendix 7.3.1 Newly eligible patients only 34](#_Toc106359305)

[Appendix 7.3.2 Existing patients Only - assumes a 5-year program to treat all existing (2019-20) eligible patients 35](#_Toc106359306)

[Appendix 7.3.3 Existing patients Only - assumes a 5-year program to treat all existing (2019-20) eligible patients 36](#_Toc106359307)

[Appendix 8: Costs 37](#_Toc106359308)

[Appendix 8.1 Total (Combined cost of private and public sector) 37](#_Toc106359309)

[Appendix 8.1.1 Newly eligible patients only 37](#_Toc106359310)

[Appendix 8.1.2 Existing patients Only - assumes a 5-year program to treat all existing (2019-20) eligible patients 39](#_Toc106359311)

[Appendix 8.1.3 Treating New Eligible patients and a 5-year program to treat existing eligible patients (as of 2019-20) 42](#_Toc106359312)

[Appendix 8.2 Private sector 44](#_Toc106359313)

[Appendix 8.2.1 Newly eligible patients only 44](#_Toc106359314)

[Appendix 8.2.2 Existing patients Only - assumes a 5-year program to treat all existing (2019-20) eligible patients 46](#_Toc106359315)

[Appendix 8.2.3 Treating New Eligible patients and a 5-year program to treat existing eligible patients (as of 2019-20) 49](#_Toc106359316)

[Appendix 8.3 Public sector 51](#_Toc106359317)

[Appendix 8.3.1 Newly eligible patients only 51](#_Toc106359318)

[Appendix 8.3.2 Existing patients Only - assumes a 5-year program to treat all existing (2019-20) eligible patients 53](#_Toc106359319)

[Appendix 8.3.3 Treating New Eligible patients and a 5-year program to treat existing eligible patients (as of 2019-20) 56](#_Toc106359320)

[References 59](#_Toc106359321)

# Appendix 1: Methodology and Data inputs

## Appendix 1.1: Methodology in detail for the future prediction of population estimates for bariatric surgery:

**Estimating the population affected by obesity in Australia:** The population of interest in this model was Australian people aged between 18-70 years old, having body mass index (BMI) ≥30. As baseline population for a single year, data was extracted for obesity sub classes (i.e., obesity class I, II and III) in this age group from the Australian Bureau of Statistics (ABS) 2017-18 National Health Survey [1].

Hays et al combined micro-simulation and survey estimation techniques to estimate likely rates of obesity in Australia to 2025 [2]. These authors kindly provided us the inputs to their epidemiological model to allow us to calculate the compound annual growth rate (CAGR) using their population estimates from 2017 to 2025 for obesity classes I, II, and III. As our base year for the population is 2017-18, we used 2017 onwards in Hays et al’s estimates to calculate the CAGR.

$$Cumulative average growth rate=\left( \frac{End value}{Start value} \right)^{\left( \frac{1}{years} \right) -1}$$

**Estimating the population eligible for primary bariatric surgery:** Sharman et al’s eligibility estimates [3] and the ANZMOSS eligibility criteria [4] were separately applied to the total obese population to estimate the eligible patients in each obesity class until 2029-30 (see Appendix 1.2 for data inputs). The eligibility criteria according to Sharman et al and ANZMOSS are shown in Table 1.

The eligible population using the prevalence rates reported by Sharman et al was separately calculated for each obesity class based on the eligibility recommendations made by the National Health and Medical Research Council guidelines for the management of overweight and obesity. We assumed that the proportion of eligible population in obesity class I, II and III would remain the same across the years of our analysis period.

The estimated eligible population based on the ANZMOSS eligibility criteria incorporates the Edmonton Obesity Staging System (EOSS) classification (Table 1), which was not used by Sharman and the NHMRC. For patients affected by Obesity class I with poorly controlled T2DM (for <10 years or with favourable C-peptide level which is poorly controlled with medication), we used Sharman et al’s original prevalence rate, as the ANZMOSS recommendation did not include an EOSS score for this group [3]. Prevalence rates of obesity by EOSS classification were used to estimate the number of eligible patients for obese class II and III with corresponding EOSS scores. In the absence of Australian data, we used prevalence rates by EOSS taken from a US analytical observational study that used data from the National Health and Human Nutrition Examination Surveys (NHANES) to examine the distribution of EOSS in a US population [5]. We used a web-based software ‘WebPlotDigitizer’ to calibrate the bar charts available in this study [6]. Other studies either did not report EOSS by obesity class, or in the case of a Brazilian study, reported an even higher prevalence [7].

**Patients becoming newly eligible for primary bariatric surgery:** We estimated the annual flow of patients becoming newly eligible for primary bariatric surgery each year. The difference in the number of obese people between two years was considered as the newly eligible population (i.e., exit from the population affected by obesity due to death or weight loss was ignored for simplicity).

**Estimating likely uptake of bariatric surgery:** Based on the literature [4, 8, 9] on patient perceptions, three potential levels of uptake of bariatric surgery were applied to the eligible population (20% / 35% / 75%). We assumed that uptake levels would be the same across all obese classes.

## Appendix 1.2: Data inputs and parameters

| **Input** | **Value** | **Source** |
| --- | --- | --- |
| Eligible Class I with poorly controlled T2DM | 0.4% of obese Australian adult population | Sharman et al., 2018[3] |
| Eligible Class II with at least one comorbidity | 14% of obese Australian adult population | Sharman et al., 2018 [3] |
| Eligible Class III | 11.8% of Australian adult population affected by obesity | Sharman et al., 2018 [3] |
| Eligible Class II with EOSS 2 and 3 | 80.9% | Padwad et al., 2010 [5] |
| Eligible Class III with EOSS 1 to 3 | 97.9% | Padwad et al., 2010 [5] |
| Uptake rate of bariatric surgery | 20% | Sarwer et al., 2013 [9] |
| Uptake rate of bariatric surgery | 35% | Lee et al., 2020 [8] |
| Uptake rate of bariatric surgery | 75% | ANZMOSS report [4] |
| Proportion of population with Private Health Insurance (Hospital cover) | 46% | AIHW Australia's Health 2020 [10] |
| The number of total bariatric surgery procedures in 2018-19 | 41,534 | AIHW [11] |
| The number of total bariatric surgery procedures in 2018-19 in private sector | 38,512 | AIHW [11] |
| The number of total bariatric surgery procedures in 2018-19 in public sector | 3,022 | AIHW [11] |

## Appendix 1.3: Data inputs and parameters for Cost and revisional surgeries

| **Parameters** | **Value** | **Reference** |
| --- | --- | --- |
| Cost of index LAGB (in 2018-19) | $ 10,049.00 | Xia et al., 2021 [12] |
| Cost of index SG (in 2018-19) | $ 12,632.00 | Xia et al., 2021 [12] |
| Cost of index RYGB (in 2018-19) | $ 15,041.00 | Xia et al., 2021 [12] |
| LAGB Revisions surgery costs (in 2018-19) | $ 12,529.37 | Estimated using Cost analysis (see appendix 1.3) |
| SG Revisions surgery costs (in 2018-19) | $ 13,576.46 | Estimated using Cost analysis (see appendix 1.3) |
| RYGB Revisions surgery costs (in 2018-19) | $ 15,186.85 | Estimated using Cost analysis (see appendix 1.3) |
| Reoperation rate of LAGB | 32.35% | Estimated using Cost analysis (see appendix 1.3) |
| Reoperation rate of SG | 0.87% | Estimated using Cost analysis (see appendix 1.3) |
| Reoperation rate of RYGB | 2.40% | Estimated using Cost analysis (see appendix 1.3) |
| Proportion of LAGB | 13.98% | Calculated using the data found from Morgan et al. The total number of index LAGB over the sum of index LAGB, SG and RYGB [13] |
| Proportion of SG | 82.50% | Calculated using the data found from Morgan et al. The total number of index SG over the sum of index LAGB, SG and RYGB [13] |
| Proportion of RYGB | 3.52% | Calculated using the data found from Morgan et al. The total number of index RYGBover the sum of index LAGB, SG and RYGB [13] |
| Frequency distribution of LAGB | 1 Reoperation: 77.99%  2 Reoperation: 18.90%  3 Reoperation: 2.66%  4 Reoperation: 0.30%  5 Reoperation: 0.12%  6 Reoperation: 0.02% | Recalculated by dividing the number of follow-up surgeries (follow-up 1-6) over the total number of revisional (follow-up 1-6; excluding the index surgery). Numbers are based on Alteri’s frequency distribution Table 1 [14] |
| Frequency distribution of SG | 1 Reoperation: 97.70%  2 Reoperation: 2.30% | Recalculated by dividing the number of follow-up surgeries (follow-up 1-6) over the total number of revisional (follow-up 1-6; excluding the index surgery). Numbers are based on Alteri’s frequency distribution Table 1 [14] |
| Frequency distribution of RYGB | 1 Reoperation: 89.43%  2 Reoperation: 9.03%  3 Reoperation: 1.08%  4 Reoperation: 0.45% | Recalculated by dividing the number of follow-up surgeries (follow-up 1-6) over the total number of revisional (follow-up 1-6; excluding the index surgery). Numbers are based on Alteri’s frequency distribution Table 1 [14] |

## Appendix 1.4: Data inputs and parameters for Cost Analysis

| **Laparoscopic Adjustable Gastric Banding** | | | | | |
| --- | --- | --- | --- | --- | --- |
| **Reoperation procedure** | **N** | **Rate** | **Mean cost** | **Mean Cost X Rate** | **Reference of Mean Costs** |
| Gastric band insertion | 13 | 0.98% | $12,410.77 | $121.95 | Campbell et al., 2019 [15] |
| Gastric band adjustment | 103 | 7.79% | $12,410.77 | $966.22 | Campbell et al., 2019 [15] |
| Gastric band removal | 225 | 17.01% | $12,410.77 | $2,110.68 | Campbell et al., 2019 [15] |
| Sleeve gastrectomy | 63 | 4.76% | $12,632.00 | $601.52 | Xia et al 2019 [12] |
| Gastric bypass any method | 14 | 1.06% | $15,041.00 | $159.16 | Xia et al 2019 [12] |
| Bariatric surgery reversal | 4 | 0.30% | $12,410.77 | $37.52 | Campbell et al., 2019 [15] |
| Bariatric surgery miscellaneous | 6 | 0.45% | $12,410.77 | $56.28 | Campbell et al., 2019 [15] |
| Sum |  | 32.35% |  | $4,053.34 |  |
| **Prevalence Rate of AE to be included in the model** |  | **32.35%** |  |  |  |
| Cost of revision for LAGB |  |  |  | **$12,529.37** |  |

*_Notes: N= Bariatric reoperations due to obesity or diabetes mellitus, mechanical device adjustment, complications, post-surgical complications following index bariatric surgery (ref: Morgan), %= N over the total number of index bariatric procedures (n=1323) [13]._*

| **Sleeve Gastrectomy** | | | | | |
| --- | --- | --- | --- | --- | --- |
| **Reoperation procedure** | **N** | **%** | **Mean cost** | **Cost X Rate** | **Reference** |
| Gastric band insertion | 4 | 0.05% | $12,410.77 | $6.36 | Campbell et al., 2019 [15] |
| Gastric band removal | 17 | 0.22% | $12,410.77 | $27.02 | Campbell et al., 2019 [15] |
| Sleeve gastrectomy | 10 | 0.13% | $12,632.00 | $16.18 | Xia et al 2019 [12] |
| Biliopancreatic diversion | 1 | 0.01% | $18,060.16 | $2.31 | IHPA Round 23 Item K10Z (direct costs) [16] |
| Gastric bypass any method | 25 | 0.32% | $15,041.00 | $48.16 | Xia et al 2019 [12] |
| Gastric reduction | 1 | 0.01% | $18,060.16 | $2.31 | IHPA Round 23 Item K10Z (direct costs) [16] |
| Bariatric surgery miscellaneous | 10 | 0.13% | $12,410.77 | $15.89 | Campbell et al., 2019 [15] |
| **Prevalence Rate of AE to be included in the model** |  | **0.87%** |  | $118.24 |  |
| **Cost of revision for LAGB** |  |  |  | **$13,576.46** |  |

*_Notes: N= Bariatric reoperations due to obesity or diabetes mellitus, mechanical device adjustment, complications, post-surgical complications following index bariatric surgery (ref: Morgan), %= N over the total number of index bariatric procedures (n=7808) [13]_*

| **Roux-en-Y Gastric Bypass** | | | | | |
| --- | --- | --- | --- | --- | --- |
| **Reoperation procedure** | **N** | **%** | **Mean cost** | **Cost x Rate** | **Reference** |
| Gastric band adjustment | 2 | 0.60% | $12,410.77 | $74.54 | Campbell et al., 2019 [15] |
| Gastric bypass any method | 2 | 0.60% | $15,041.00 | $90.34 | Xia et al 2019 [12] |
| Gastric reduction | 3 | 0.90% | $18,060.16 | $162.70 | IHPA Round 23 Item K10Z (direct costs) [16] |
| Bariatric surgery miscellaneous | 1 | 0.30% | $12,410.77 | $37.27 | Campbell et al., 2019 [15] |
| **Prevalence Rate of AE to be included in the model** |  | 2.40% |  | $364.85 |  |
|  |  |  |  | $15,186.85 |  |

*_Notes: N= Bariatric reoperations due to obesity or diabetes mellitus, mechanical device adjustment, complications, post-surgical complications following index bariatric surgery , %= N over the total number of index bariatric procedures (n=333) [13]_*

# Appendix 2 Capacity model (Combined Private and Public)

## Appendix 2.1 Bariatric surgical capacity and Description of Scenarios

The historical number of total bariatric surgery procedures were obtained from AIHW. The most recent capacity reported was for the year 2019-20, however, the number of procedures performed that year was constrained by the COVID-19 pandemic. Therefore, the number of bariatric surgeries conducted in the previous year (2018-19) was considered as reflecting current system capacity, which was 41,534. This included 39,332 procedures in the private sector and 3,022 in the public sector[17]. The number of primary procedures and revisions were calculated based on Morgan et al’s estimates [13].

*Scenario 1: Newly eligible patients only*

Taking 2019-20 as the starting year, primary bariatric surgery is offered only to newly eligible patients (in the year in which the first become eligible) in this scenario, considering possible uptake rates of 25%, 30% and 75% of eligible patients for each year until 2029-30. Based on the proportion of population with private health insurance, newly eligible patients were separated into public and private demand incorporating the same uptake levels. Based on the actual number of primary bariatric procedures in year 2019-20, the gap between demand and supply in public sector, private sector and in both sectors combined were modelled for newly eligible patients for each year from 2019-20 to 2029-30.

*Scenario 2: Existing patients only - assuming a 5-year program to treat all existing eligible patients (as of 2019-20)*

The total number of eligible people for a primary bariatric procedure observed in the year 2019-20 was considered as the baseline population. This scenario envisages treating all of them over a 5-year period, starting from year 2022-23. The same potential uptake rates of 25%, 30%, and 75% were included. Current surgical capacity as a percentage of total eligible patients was then calculated.

*Scenario 3: Treating all newly eligible patients and a 5-year program to treat existing patients*

The number of people newly eligible for primary procedure each year was added to the already existing eligible population shown in scenario 2 over the 5-year period at 25%, 30% and 75% uptake level in both private and public sector, starting from year 2022-23.

## Appendix 2.2 Capacity model

| **Demand** | **2022-23** | **2023-24** | **2024-25** | **2025-26** | **2026-27** | **2027-28** | **2028-29** | **2029-30** |
| --- | --- | --- | --- | --- | --- | --- | --- | --- |
| *Scenario 1. Newly eligible patients only* | | | | | | | | |
| Total New patients | 48867 | 50262 | 51698 | 53177 | 54701 | 56270 | 57886 | 59551 |
| 20% uptake | 9773 | 10052 | 10340 | 10635 | 10940 | 11254 | 11577 | 11910 |
| 35% uptake | 17103 | 17592 | 18094 | 18612 | 19145 | 19694 | 20260 | 20843 |
| 75% uptake | 36650 | 37696 | 38773 | 39883 | 41025 | 42202 | 43415 | 44664 |
| Private Demand | | | | | | | | |
| 20% uptake | 4496 | 4624 | 4756 | 4892 | 5032 | 5177 | 5326 | 5479 |
| 35% uptake | 7868 | 8092 | 8323 | 8562 | 8807 | 9059 | 9320 | 9588 |
| 75% uptake | 16859 | 17340 | 17836 | 18346 | 18872 | 19413 | 19971 | 20545 |
| Public Demand | | | | | | | | |
| 20% uptake | 5278 | 5428 | 5583 | 5743 | 5908 | 6077 | 6252 | 6432 |
| 35% uptake | 9236 | 9499 | 9771 | 10050 | 10338 | 10635 | 10941 | 11255 |
| 75% uptake | 19791 | 20356 | 20938 | 21537 | 22154 | 22789 | 23444 | 24118 |
| *Scenario 2. Existing patients only - assumes a 5-year program to treat all existing (2019-20) eligible patients* | | | | | | | | |
| Total Existing patients | 341343 | 341343 | 341343 | 341343 | 341343 | - | - | - |
| 20% uptake | 68269 | 68269 | 68269 | 68269 | 68269 | - | - | - |
| 35% uptake | 119470 | 119470 | 119470 | 119470 | 119470 | - | - | - |
| 75% uptake | 256007 | 256007 | 256007 | 256007 | 256007 | - | - | - |
| Private Demand | | | | | | | | |
| 20% uptake | 31404 | 31404 | 31404 | 31404 | 31404 | - | - | - |
| 35% uptake | 54956 | 54956 | 54956 | 54956 | 54956 | - | - | - |
| 75% uptake | 117763 | 117763 | 117763 | 117763 | 117763 | - | - | - |
| Public Demand | | | | | | | | |
| 20% uptake | 36865 | 36865 | 36865 | 36865 | 36865 | - | - | - |
| 35% uptake | 64514 | 64514 | 64514 | 64514 | 64514 | - | - | - |
| 75% uptake | 138244 | 138244 | 138244 | 138244 | 138244 | - | - | - |
| *Scenario 3. Treating New Eligible patients and a 5-year program to treat existing eligible patients* | | | | | | | | |
| Total patients | 390210 | 391604 | 393041 | 394520 | 396043 | 56270 | 57886 | 59551 |
| 20% uptake | 78042 | 78321 | 78608 | 78904 | 79209 | 11254 | 11577 | 11910 |
| 35% uptake | 136573 | 137062 | 137564 | 138082 | 138615 | 19694 | 20260 | 20843 |
| 75% uptake | 292657 | 293703 | 294780 | 295890 | 297033 | 42202 | 43415 | 44664 |
| Private Demand | | | | | | | | |
| 20% uptake | 35899 | 36028 | 36160 | 36296 | 36436 | 5177 | 5326 | 5479 |
| 35% uptake | 62824 | 63048 | 63280 | 63518 | 63763 | 9059 | 9320 | 9588 |
| 75% uptake | 134622 | 135103 | 135599 | 136109 | 136635 | 19413 | 19971 | 20545 |
| Public Demand | | | | | | | | |
| 20% uptake | 42143 | 42293 | 42448 | 42608 | 42773 | 6077 | 6252 | 6432 |
| 35% uptake | 73750 | 74013 | 74285 | 74564 | 74852 | 10635 | 10941 | 11255 |
| 75% uptake | 158035 | 158600 | 159181 | 159781 | 160398 | 22789 | 23444 | 24118 |
| **Capacity** | | | | | | | | |
| Total capacity for primary surgery | 39322 | 39322 | 39322 | 39322 | 39322 | 39322 | 39322 | 39322 |
| Public sector | 2861 | 2861 | 2861 | 2861 | 2861 | 2861 | 2861 | 2861 |
| Private sector | 36461 | 36461 | 36461 | 36461 | 36461 | 36461 | 36461 | 36461 |

_“-“: Not Applicable_

# Appendix 3: Decision tree


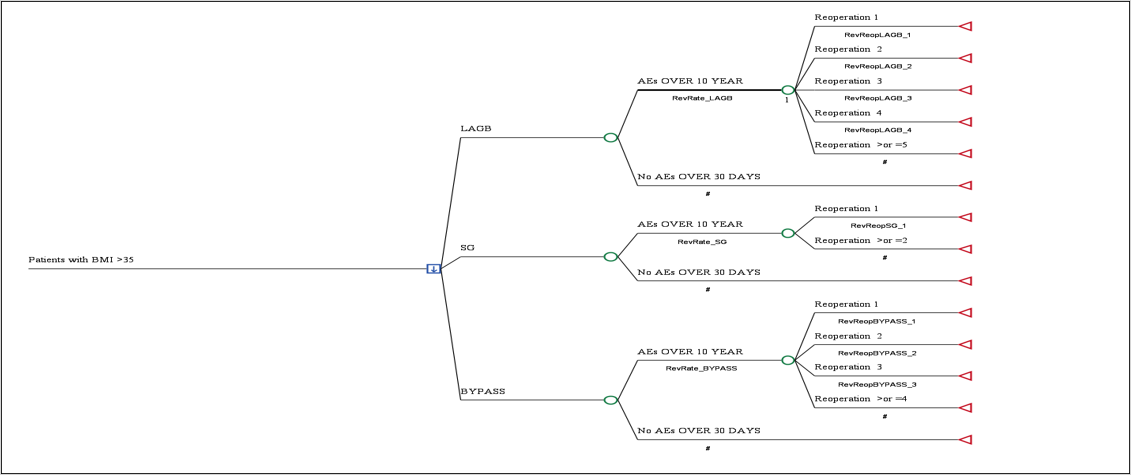


_Notes: LAGB= Laparoscopic Adjustable Gastric Banding, SG =Sleeve Gastrectomy, AE= Adverse events, RevRate=Revision rate_

The model structure consists of two health states: patients suffer with or without adverse events requiring subsequent reoperations. The number of reoperations depends on the type of primary surgery. According to Alteri et al [14], some patients who underwent LAGB had up to six reoperations, while a proportion of patients who underwent SG and RYGB had up to two and five revisions respectively. The revisions arising from the primary surgery were pragmatically distributed equally over a 10-year period [13, 14]. Mean time to the subsequent procedure was reported as 3.8 years (SD 1.5-6.1) for LAGB, 3 (SD 0.8-5.2) for SG, and 3.6 (SD 0.7-6.5) [14]. In addition, according to a Australian population cohort study, the median time for a 25% of the cohort was 75.1 months, five months, 30 months and 14 months for the first, second, third and fourth bariatric reoperations respectively [13].

**Data inputs**

The reoperation rates and adverse events rates were extracted from the same population cohort study in Western Australia, and these were adjusted excluding reoperations due to cosmetic reasons [13]. The frequency distribution of follow-up surgeries was obtained from a retrospective analysis over a ten-year period [14].

The costs for the primary LAGB, SG and RYGB were sourced from a study investigating the direct medical costs of bariatric surgery in the Tasmanian public health system [12]. Meanwhile, a cost analysis was undertaken to estimate the cost of the adverse events arising from the primary bariatric surgery. In the cost analysis, we weighted the rates of adverse events due to the primary surgery to the costs of primary and revision surgeries. The rates of adverse events were obtained from the Western Australia cohort study and the reoperation costs were sourced from a recently published study in Australia [12], and the Independent Hospital Pricing Authority [16]. Some costs such as revisions and reversal costs were inflated according to the government final consumption expenditure on hospital and nursing homes [18] and all costs were presented in Australian dollars with base year of 2018-19.

The likely demand for scenarios 1, 2 and 3 were run in the model. The result of this analysis presents the total number of primary surgeries, number of revisions and the costs for private, public sector and both combined, based on the proportion of population with private health insurance. See Appendix 1 for model inputs.

# Appendix 4: Primary and Potential revisions arising from the primary surgery

## Appendix 4.1 Total for Newly eligible patients only (Combined figures of private and public sector)

| **Surgery & uptake level** | **2022-23** | **2023-24** | **2024-25** | **2025-26** | **2026-27** | **2027-28** | **2028-29** | **2029-30** |
| --- | --- | --- | --- | --- | --- | --- | --- | --- |
| Primary surgery 20% uptake | | | | | | | | |
| LAGB | 1,366 | 1,405 | 1,445 | 1,487 | 1,529 | 1,573 | 1,618 | 1,665 |
| SG | 8,063 | 8,293 | 8,530 | 8,774 | 9,026 | 9,285 | 9,551 | 9,826 |
| RYGB | 344 | 354 | 364 | 374 | 385 | 396 | 407 | 419 |
| Potential Revisions 20% uptake | | | | | | | | |
| LAGB | 556 | 572 | 588 | 605 | 622 | 640 | 658 | 677 |
| SG | 72 | 74 | 76 | 78 | 80 | 83 | 85 | 88 |
| RYGB | 9 | 10 | 10 | 10 | 10 | 11 | 11 | 11 |
| Primary surgery 35% uptake | | | | | | | | |
| LAGB | 2,391 | 2,459 | 2,529 | 2,602 | 2,676 | 2,753 | 2,832 | 2,914 |
| SG | 14,111 | 14,513 | 14,928 | 15,355 | 15,795 | 16,248 | 16,715 | 17,196 |
| RYGB | 602 | 619 | 637 | 655 | 674 | 693 | 713 | 733 |
| Potential Revisions 35% uptake | | | | | | | | |
| LAGB | 972 | 1,000 | 1,029 | 1,058 | 1,089 | 1,120 | 1,152 | 1,185 |
| SG | 126 | 129 | 133 | 137 | 141 | 145 | 149 | 153 |
| RYGB | 16 | 17 | 17 | 18 | 18 | 19 | 19 | 20 |
| Primary surgery 75% uptake | | | | | | | | |
| LAGB | 5,123 | 5,270 | 5,420 | 5,575 | 5,735 | 5,900 | 6,069 | 6,244 |
| SG | 30,237 | 31,100 | 31,989 | 32,904 | 33,847 | 34,818 | 35,818 | 36,848 |
| RYGB | 1,290 | 1,326 | 1,364 | 1,403 | 1,444 | 1,485 | 1,528 | 1,572 |
| Potential Revisions 75% uptake | | | | | | | | |
| LAGB | 2,084 | 2,143 | 2,205 | 2,268 | 2,333 | 2,400 | 2,468 | 2,539 |
| SG | 269 | 277 | 285 | 293 | 302 | 310 | 319 | 328 |
| RYGB | 35 | 36 | 37 | 38 | 39 | 40 | 41 | 42 |

*_Note: Potential revisions are distributed in a 10-year period (see Appendix 6)_*

## Appendix 4.2 Total Existing patients Only - assumes a 5-year program to treat all existing (2019-20) eligible patients

| **Surgery & uptake level** | **2022-23** | **2023-24** | **2024-25** | **2025-26** | **2026-27** | **2027-28** | **2028-29** | **2029-30** |
| --- | --- | --- | --- | --- | --- | --- | --- | --- |
| Primary surgery 20% uptake | | | | | | | | |
| LAGB | 9,543 | 9,543 | 9,543 | 9,543 | 9,543 | - | - | - |
| SG | 56,323 | 56,323 | 56,323 | 56,323 | 56,323 | - | - | - |
| RYGB | 2,402 | 2,402 | 2,402 | 2,402 | 2,402 | - | - | - |
| Potential Revisions 20% uptake | | | | | | | | |
| LAGB | 3,882 | 3,882 | 3,882 | 3,882 | 3,882 | - | - | - |
| SG | 502 | 502 | 502 | 502 | 502 | - | - | - |
| RYGB | 65 | 65 | 65 | 65 | 65 | - | - | - |
| Primary surgery 35% uptake | | | | | | | | |
| LAGB | 16,701 | 16,701 | 16,701 | 16,701 | 16,701 | - | - | - |
| SG | 98,565 | 98,565 | 98,565 | 98,565 | 98,565 | - | - | - |
| RYGB | 4,204 | 4,204 | 4,204 | 4,204 | 4,204 | - | - | - |
| Potential Revisions 35% uptake | | | | | | | | |
| LAGB | 6,793 | 6,793 | 6,793 | 6,793 | 6,793 | - | - | - |
| SG | 878 | 878 | 878 | 878 | 878 | - | - | - |
| RYGB | 114 | 114 | 114 | 114 | 114 | - | - | - |
| Primary surgery 75% uptake | | | | | | | | |
| LAGB | 35,788 | 35,788 | 35,788 | 35,788 | 35,788 | - | - | - |
| SG | 211,211 | 211,211 | 211,211 | 211,211 | 211,211 | - | - | - |
| RYGB | 9,008 | 9,008 | 9,008 | 9,008 | 9,008 | - | - | - |
| Potential Revisions 75% uptake | | | | | | | | |
| LAGB | 14,556 | 14,556 | 14,556 | 14,556 | 14,556 | - | - | - |
| SG | 1,882 | 1,882 | 1,882 | 1,882 | 1,882 | - | - | - |
| RYGB | 244 | 244 | 244 | 244 | 244 | - | - | - |

_“-“: Not Applicable_

## Appendix 4.3 total Newly Eligible patients plus a 5-year program to treat existing eligible patients (as of 2019-20)

| **Surgery & uptake** | **2022-23** | **2023-24** | **2024-25** | **2025-26** | **2026-27** | **2027-28** | **2028-29** | **2029-30** |
| --- | --- | --- | --- | --- | --- | --- | --- | --- |
| Primary surgery 20% uptake | | | | | | | | |
| LAGB | 10,910 | 10,949 | 10,989 | 11,030 | 11,073 | 1,573 | 1,618 | 1,665 |
| SG | 64,386 | 64,616 | 64,853 | 65,097 | 65,349 | 9,285 | 9,551 | 9,826 |
| RYGB | 2,746 | 2,756 | 2,766 | 2,776 | 2,787 | 396 | 407 | 419 |
| Potential Revisions 20% uptake | | | | | | | | |
| LAGB | 4,437 | 4,453 | 4,469 | 4,486 | 4,504 | 640 | 658 | 677 |
| SG | 574 | 576 | 578 | 580 | 582 | 83 | 85 | 88 |
| RYGB | 74 | 75 | 75 | 75 | 75 | 11 | 11 | 11 |
| Primary surgery 35% uptake | | | | | | | | |
| LAGB | 19,092 | 19,160 | 19,230 | 19,303 | 19,377 | 2,753 | 2,832 | 2,914 |
| SG | 112,676 | 113,079 | 113,493 | 113,921 | 114,360 | 16,248 | 16,715 | 17,196 |
| RYGB | 4,805 | 4,823 | 4,840 | 4,859 | 4,877 | 693 | 713 | 733 |
| Potential Revisions 35% uptake | | | | | | | | |
| LAGB | 7,765 | 7,793 | 7,822 | 7,851 | 7,881 | 1,120 | 1,152 | 1,185 |
| SG | 1,004 | 1,007 | 1,011 | 1,015 | 1,019 | 145 | 149 | 153 |
| RYGB | 130 | 130 | 131 | 131 | 132 | 19 | 19 | 20 |
| Primary surgery 75% uptake | | | | | | | | |
| LAGB | 40,911 | 41,058 | 41,208 | 41,363 | 41,523 | 5,900 | 6,069 | 6,244 |
| SG | 241,448 | 242,311 | 243,200 | 244,115 | 245,058 | 34,818 | 35,818 | 36,848 |
| RYGB | 10,297 | 10,334 | 10,372 | 10,411 | 10,451 | 1,485 | 1,528 | 1,572 |
| Potential Revisions 75% uptake | | | | | | | | |
| LAGB | 16,640 | 16,699 | 16,760 | 16,824 | 16,889 | 2,400 | 2,468 | 2,539 |
| SG | 2,151 | 2,159 | 2,167 | 2,175 | 2,183 | 310 | 319 | 328 |
| RYGB | 278 | 279 | 280 | 282 | 283 | 40 | 41 | 42 |

# Appendix 5: Primary and Potential revisions arising from the primary surgery in the Private sector

## Appendix 5.1 Newly eligible patients only

| **Surgery & uptake** | **2022-23** | **2023-24** | **2024-25** | **2025-26** | **2026-27** | **2027-28** | **2028-29** | **2029-30** |
| --- | --- | --- | --- | --- | --- | --- | --- | --- |
| Primary surgery 20% uptake | | | | | | | | |
| LAGB | 628 | 646 | 665 | 684 | 704 | 724 | 744 | 766 |
| SG | 3,709 | 3,815 | 3,924 | 4,036 | 4,152 | 4,271 | 4,394 | 4,520 |
| RYGB | 158 | 163 | 167 | 172 | 177 | 182 | 187 | 193 |
| Potential Revisions 20% uptake | | | | | | | | |
| LAGB | 256 | 263 | 270 | 278 | 286 | 294 | 303 | 312 |
| SG | 33 | 34 | 35 | 36 | 37 | 38 | 39 | 40 |
| RYGB | 4 | 4 | 5 | 5 | 5 | 5 | 5 | 5 |
| Primary surgery 35% uptake | | | | | | | | |
| LAGB | 1,100 | 1,131 | 1,164 | 1,197 | 1,231 | 1,266 | 1,303 | 1,340 |
| SG | 6,491 | 6,676 | 6,867 | 7,063 | 7,266 | 7,474 | 7,689 | 7,910 |
| RYGB | 277 | 285 | 293 | 301 | 310 | 319 | 328 | 337 |
| Potential Revisions 35% uptake | | | | | | | | |
| LAGB | 447 | 460 | 473 | 487 | 501 | 515 | 530 | 545 |
| SG | 58 | 59 | 61 | 63 | 65 | 67 | 69 | 70 |
| RYGB | 7 | 8 | 8 | 8 | 8 | 9 | 9 | 9 |
| Primary surgery 75% uptake | | | | | | | | |
| LAGB | 2,357 | 2,424 | 2,493 | 2,565 | 2,638 | 2,714 | 2,792 | 2,872 |
| SG | 13,909 | 14,306 | 14,715 | 15,136 | 15,570 | 16,016 | 16,476 | 16,950 |
| RYGB | 593 | 610 | 628 | 646 | 664 | 683 | 703 | 723 |
| Potential Revisions 75% uptake | | | | | | | | |
| LAGB | 959 | 986 | 1,014 | 1,043 | 1,073 | 1,104 | 1,135 | 1,168 |
| SG | 124 | 127 | 131 | 135 | 139 | 143 | 147 | 151 |
| RYGB | 16 | 16 | 17 | 17 | 18 | 18 | 19 | 20 |

## Appendix 5.2 Existing patients Only - assumes a 5-year program to treat all existing eligible patients (2019-20)

| Surgery & uptake | **2022-23** | **2023-24** | **2024-25** | **2025-26** | **2026-27** | **2027-28** | **2028-29** | **2029-30** |
| --- | --- | --- | --- | --- | --- | --- | --- | --- |
| Primary surgery 20% uptake | | | | | | | | |
| LAGB | 4,390 | 4,390 | 4,390 | 4,390 | 4,390 | - | - | - |
| SG | 25,909 | 25,909 | 25,909 | 25,909 | 25,909 | - | - | - |
| RYGB | 1,105 | 1,105 | 1,105 | 1,105 | 1,105 | - | - | - |
| Potential Revisions 20% uptake | | | | | | | | |
| LAGB | 1,786 | 1,786 | 1,786 | 1,786 | 1,786 | - | - | - |
| SG | 231 | 231 | 231 | 231 | 231 | - | - | - |
| RYGB | 30 | 30 | 30 | 30 | 30 | - | - | - |
| Primary surgery 35% uptake | | | | | | | | |
| LAGB | 7,682 | 7,682 | 7,682 | 7,682 | 7,682 | - | - | - |
| SG | 45,340 | 45,340 | 45,340 | 45,340 | 45,340 | - | - | - |
| RYGB | 1,934 | 1,934 | 1,934 | 1,934 | 1,934 | - | - | - |
| Potential Revisions 35% uptake | | | | | | | | |
| LAGB | 3,125 | 3,125 | 3,125 | 3,125 | 3,125 | - | - | - |
| SG | 404 | 404 | 404 | 404 | 404 | - | - | - |
| RYGB | 52 | 52 | 52 | 52 | 52 | - | - | - |
| Primary surgery 75% uptake | | | | | | | | |
| LAGB | 16,462 | 16,462 | 16,462 | 16,462 | 16,462 | - | - | - |
| SG | 97,157 | 97,157 | 97,157 | 97,157 | 97,157 | - | - | - |
| RYGB | 4,144 | 4,144 | 4,144 | 4,144 | 4,144 | - | - | - |
| Potential Revisions 75% uptake | | | | | | | | |
| LAGB | 6,696 | 6,696 | 6,696 | 6,696 | 6,696 | - | - | - |
| SG | 866 | 866 | 866 | 866 | 866 | - | - | - |
| RYGB | 112 | 112 | 112 | 112 | 112 | - | - | - |

_“-“: Not Applicable_

## Appendix 5.3 Treating New Eligible patients plus a 5-year program to treat existing eligible patients (as of 2019-20)

| **Surgery & uptake** | **2022-23** | **2023-24** | **2024-25** | **2025-26** | **2026-27** | **2027-28** | **2028-29** | **2029-30** |
| --- | --- | --- | --- | --- | --- | --- | --- | --- |
| Primary surgery 20% uptake | | | | | | | | |
| LAGB | 5,018 | 5,036 | 5,055 | 5,074 | 5,093 | 724 | 744 | 766 |
| SG | 29,618 | 29,724 | 29,833 | 29,945 | 30,060 | 4,271 | 4,394 | 4,520 |
| RYGB | 1,263 | 1,268 | 1,272 | 1,277 | 1,282 | 182 | 187 | 193 |
| Potential Revisions 20% uptake | | | | | | | | |
| LAGB | 2,041 | 2,048 | 2,056 | 2,064 | 2,072 | 294 | 303 | 312 |
| SG | 264 | 265 | 266 | 267 | 268 | 38 | 39 | 40 |
| RYGB | 34 | 34 | 34 | 35 | 35 | 5 | 5 | 5 |
| Primary surgery 35% uptake | | | | | | | | |
| LAGB | 8,782 | 8,814 | 8,846 | 8,879 | 8,914 | 1,266 | 1,303 | 1,340 |
| SG | 51,831 | 52,016 | 52,207 | 52,403 | 52,606 | 7,474 | 7,689 | 7,910 |
| RYGB | 2,211 | 2,218 | 2,227 | 2,235 | 2,244 | 319 | 328 | 337 |
| Potential Revisions 35% uptake | | | | | | | | |
| LAGB | 3,572 | 3,585 | 3,598 | 3,611 | 3,625 | 515 | 530 | 545 |
| SG | 462 | 463 | 465 | 467 | 469 | 67 | 69 | 70 |
| RYGB | 60 | 60 | 60 | 60 | 61 | 9 | 9 | 9 |
| Primary surgery 75% uptake | | | | | | | | |
| LAGB | 18,819 | 18,887 | 18,956 | 19,027 | 19,101 | 2,714 | 2,792 | 2,872 |
| SG | 111,066 | 111,463 | 111,872 | 112,293 | 112,727 | 16,016 | 16,476 | 16,950 |
| RYGB | 4,737 | 4,754 | 4,771 | 4,789 | 4,808 | 683 | 703 | 723 |
| Potential Revisions 75% uptake | | | | | | | | |
| LAGB | 7,654 | 7,682 | 7,710 | 7,739 | 7,769 | 1,104 | 1,135 | 1,168 |
| SG | 990 | 993 | 997 | 1,000 | 1,004 | 143 | 147 | 151 |
| RYGB | 128 | 129 | 129 | 130 | 130 | 18 | 19 | 20 |

# Appendix 6: Primary and Potential revisions arising from the primary surgery in the public sector

## Appendix 6.1 Newly eligible patients only

| **Surgery & uptake** | **2022-23** | **2023-24** | **2024-25** | **2025-26** | **2026-27** | **2027-28** | **2028-29** | **2029-30** |
| --- | --- | --- | --- | --- | --- | --- | --- | --- |
| Primary surgery 20% uptake | | | | | | | | |
| LAGB | 738 | 759 | 781 | 803 | 826 | 850 | 874 | 899 |
| SG | 4,354 | 4,478 | 4,606 | 4,738 | 4,874 | 5,014 | 5,158 | 5,306 |
| RYGB | 186 | 191 | 196 | 202 | 208 | 214 | 220 | 226 |
| Potential Revisions 20% uptake | | | | | | | | |
| LAGB | 300 | 309 | 317 | 327 | 336 | 346 | 355 | 366 |
| SG | 39 | 40 | 41 | 42 | 43 | 45 | 46 | 47 |
| RYGB | 5 | 5 | 5 | 5 | 6 | 6 | 6 | 6 |
| Primary surgery 35% uptake | | | | | | | | |
| LAGB | 1,291 | 1,328 | 1,366 | 1,405 | 1,445 | 1,487 | 1,529 | 1,573 |
| SG | 7,620 | 7,837 | 8,061 | 8,292 | 8,529 | 8,774 | 9,026 | 9,286 |
| RYGB | 325 | 334 | 344 | 354 | 364 | 374 | 385 | 396 |
| Potential Revisions 35% uptake | | | | | | | | |
| LAGB | 525 | 540 | 556 | 571 | 588 | 605 | 622 | 640 |
| SG | 68 | 70 | 72 | 74 | 76 | 78 | 80 | 83 |
| RYGB | 9 | 9 | 9 | 10 | 10 | 10 | 10 | 11 |
| Primary surgery 75% uptake | | | | | | | | |
| LAGB | 2,767 | 2,846 | 2,927 | 3,011 | 3,097 | 3,186 | 3,277 | 3,372 |
| SG | 16,328 | 16,794 | 17,274 | 17,768 | 18,277 | 18,802 | 19,342 | 19,898 |
| RYGB | 696 | 716 | 737 | 758 | 780 | 802 | 825 | 849 |
| Potential Revisions 75% uptake | | | | | | | | |
| LAGB | 1,125 | 1,157 | 1,190 | 1,225 | 1,260 | 1,296 | 1,333 | 1,371 |
| SG | 145 | 150 | 154 | 158 | 163 | 168 | 172 | 177 |
| RYGB | 19 | 19 | 20 | 20 | 21 | 22 | 22 | 23 |

## Appendix 6.2 Existing patients Only - assumes a 5-year program to treat all existing (2019-20) eligible patients

| **Surgery & uptake** | **2022-23** | **2023-24** | **2024-25** | **2025-26** | **2026-27** | **2027-28** | **2028-29** | **2029-30** |
| --- | --- | --- | --- | --- | --- | --- | --- | --- |
| Primary surgery 20% uptake | | | | | | | | |
| LAGB | 5,153 | 5,153 | 5,153 | 5,153 | 5,153 | - | - | - |
| SG | 30,414 | 30,414 | 30,414 | 30,414 | 30,414 | - | - | - |
| RYGB | 1,297 | 1,297 | 1,297 | 1,297 | 1,297 | - | - | - |
| Potential Revisions 20% uptake | | | | | | | | |
| LAGB | 2,096 | 2,096 | 2,096 | 2,096 | 2,096 | - | - | - |
| SG | 271 | 271 | 271 | 271 | 271 | - | - | - |
| RYGB | 35 | 35 | 35 | 35 | 35 | - | - | - |
| Primary surgery 35% uptake | | | | | | | | |
| LAGB | 9,019 | 9,019 | 9,019 | 9,019 | 9,019 | - | - | - |
| SG | 53,225 | 53,225 | 53,225 | 53,225 | 53,225 | - | - | - |
| RYGB | 2,270 | 2,270 | 2,270 | 2,270 | 2,270 | - | - | - |
| Potential Revisions 35% uptake | | | | | | | | |
| LAGB | 3,668 | 3,668 | 3,668 | 3,668 | 3,668 | - | - | - |
| SG | 474 | 474 | 474 | 474 | 474 | - | - | - |
| RYGB | 61 | 61 | 61 | 61 | 61 | - | - | - |
| Primary surgery 75% uptake | | | | | | | | |
| LAGB | 19,326 | 19,326 | 19,326 | 19,326 | 19,326 | - | - | - |
| SG | 114,054 | 114,054 | 114,054 | 114,054 | 114,054 | - | - | - |
| RYGB | 4,864 | 4,864 | 4,864 | 4,864 | 4,864 | - | - | - |
| Potential Revisions 75% uptake | | | | | | | | |
| LAGB | 7,860 | 7,860 | 7,860 | 7,860 | 7,860 | - | - | - |
| SG | 1,016 | 1,016 | 1,016 | 1,016 | 1,016 | - | - | - |
| RYGB | 132 | 132 | 132 | 132 | 132 | - | - | - |

_“-“: Not Applicable_

## Appendix 6.3 Treating New Eligible patients and a 5-year program to treat existing eligible patients (as of 2019-20)

| **Surgery & uptake** | **2022-23** | **2023-24** | **2024-25** | **2025-26** | **2026-27** | **2027-28** | **2028-29** | **2029-30** |
| --- | --- | --- | --- | --- | --- | --- | --- | --- |
| Primary surgery 20% uptake | | | | | | | | |
| LAGB | 5,891 | 5,912 | 5,934 | 5,956 | 5,979 | 850 | 874 | 899 |
| SG | 34,769 | 34,893 | 35,021 | 35,153 | 35,288 | 5,014 | 5,158 | 5,306 |
| RYGB | 1,483 | 1,488 | 1,494 | 1,499 | 1,505 | 214 | 220 | 226 |
| Potential Revisions 20% uptake | | | | | | | | |
| LAGB | 2,396 | 2,405 | 2,414 | 2,423 | 2,432 | 346 | 355 | 366 |
| SG | 310 | 311 | 312 | 313 | 314 | 45 | 46 | 47 |
| RYGB | 40 | 40 | 40 | 41 | 41 | 6 | 6 | 6 |
| Primary surgery 35% uptake | | | | | | | | |
| LAGB | 10,310 | 10,347 | 10,384 | 10,424 | 10,464 | 1,487 | 1,529 | 1,573 |
| SG | 60,845 | 61,062 | 61,286 | 61,517 | 61,755 | 8,774 | 9,026 | 9,286 |
| RYGB | 2,595 | 2,604 | 2,614 | 2,624 | 2,634 | 374 | 385 | 396 |
| Potential Revisions 35% uptake | | | | | | | | |
| LAGB | 4,193 | 4,208 | 4,224 | 4,240 | 4,256 | 605 | 622 | 640 |
| SG | 542 | 544 | 546 | 548 | 550 | 78 | 80 | 83 |
| RYGB | 70 | 70 | 71 | 71 | 71 | 10 | 10 | 11 |
| Primary surgery 75% uptake | | | | | | | | |
| LAGB | 22,092 | 22,171 | 22,252 | 22,336 | 22,422 | 3,186 | 3,277 | 3,372 |
| SG | 130,382 | 130,848 | 131,328 | 131,822 | 132,331 | 18,802 | 19,342 | 19,898 |
| RYGB | 5,561 | 5,580 | 5,601 | 5,622 | 5,644 | 802 | 825 | 849 |
| Potential Revisions 75% uptake | | | | | | | | |
| LAGB | 8,985 | 9,018 | 9,051 | 9,085 | 9,120 | 1,296 | 1,333 | 1,371 |
| SG | 1,162 | 1,166 | 1,170 | 1,174 | 1,179 | 168 | 172 | 177 |
| RYGB | 150 | 151 | 151 | 152 | 153 | 22 | 22 | 23 |

# Appendix 7: Distribution of Revisions Over 10-year time horizon

## Appendix 7.1 Total (private plus private sector)

### Appendix 7.1.1 Newly eligible patients only

| **Uptake** | **2022-23** | **2023-24** | **2024-25** | **2025-26** | **2026-27** | **2027-28** | **2028-29** | **2029-30** | **2030-31** | **2031-32** | **2032-33** | **2033-34** | **2034-35** | **2035-36** | **2036-37** | **2037-38** | **2038-39** | **2039-40** |
| --- | --- | --- | --- | --- | --- | --- | --- | --- | --- | --- | --- | --- | --- | --- | --- | --- | --- | --- |
| 20% uptake | | | | | | | | | | | | | | | | | | |
| LAGB | NA | 56 | 113 | 172 | 232 | 294 | 358 | 424 | 492 | 492 | 492 | 436 | 379 | 320 | 260 | 198 | 134 | 68 |
| SG | NA | 7 | 15 | 22 | 30 | 38 | 46 | 55 | 64 | 64 | 64 | 56 | 49 | 41 | 34 | 26 | 17 | 9 |
| RYGB | NA | 1 | 2 | 3 | 4 | 5 | 6 | 7 | 8 | 8 | 8 | 7 | 6 | 5 | 4 | 3 | 2 | 1 |
| 35% uptake | | | | | | | | | | | | | | | | | | |
| LAGB | NA | 97 | 197 | 300 | 406 | 515 | 627 | 742 | 861 | 861 | 861 | 763 | 663 | 560 | 455 | 346 | 234 | 119 |
| SG | NA | 13 | 26 | 39 | 52 | 67 | 81 | 96 | 111 | 111 | 111 | 99 | 86 | 72 | 59 | 45 | 30 | 15 |
| RYGB | NA | 2 | 3 | 5 | 7 | 9 | 10 | 12 | 14 | 14 | 14 | 13 | 11 | 9 | 8 | 6 | 4 | 2 |
| 75% uptake | | | | | | | | | | | | | | | | | | |
| LAGB | NA | 208 | 423 | 643 | 870 | 1103 | 1343 | 1590 | 1844 | 1844 | 1844 | 1636 | 1421 | 1201 | 974 | 741 | 501 | 254 |
| SG | NA | 27 | 55 | 83 | 112 | 143 | 174 | 206 | 238 | 238 | 238 | 211 | 184 | 155 | 126 | 96 | 65 | 33 |
| RYGB | NA | 3 | 7 | 11 | 15 | 18 | 22 | 27 | 31 | 31 | 31 | 27 | 24 | 20 | 16 | 12 | 8 | 4 |

_NA/previous year_

### Appendix 7.1.2 Existing patients Only - assumes a 5-year program to treat all existing (2019-20) eligible patients

| **uptake** | **2022-23** | **2023-24** | **2024-25** | **2025-26** | **2026-27** | **2027-28** | **2028-29** | **2029-30** | **2030-31** | **2031-32** | **2032-33** | **2033-34** | **2034-35** | **2035-36** | **2036-37** | **2037-38** | **2038-39** | **2039-40** |
| --- | --- | --- | --- | --- | --- | --- | --- | --- | --- | --- | --- | --- | --- | --- | --- | --- | --- | --- |
| 20% uptake | | | | | | | | | | | | | | | | | | |
| LAGB | NA | 388 | 776 | 1164 | 1553 | 1941 | 1941 | 1941 | 1941 | 1941 | 1941 | 1553 | 1164 | 776 | 388 | 0 | 0 | 0 |
| SG | NA | 50 | 100 | 151 | 201 | 251 | 251 | 251 | 251 | 251 | 251 | 201 | 151 | 100 | 50 | 0 | 0 | 0 |
| RYGB | NA | 6 | 13 | 19 | 26 | 32 | 32 | 32 | 32 | 32 | 32 | 26 | 19 | 13 | 6 | 0 | 0 | 0 |
| 35% uptake | | | | | | | | | | | | | | | | | | |
| LAGB | NA | 679 | 1359 | 2038 | 2717 | 3396 | 3396 | 3396 | 3396 | 3396 | 3396 | 2717 | 2038 | 1359 | 679 | 0 | 0 | 0 |
| SG | NA | 88 | 176 | 263 | 351 | 439 | 439 | 439 | 439 | 439 | 439 | 351 | 263 | 176 | 88 | 0 | 0 | 0 |
| RYGB | NA | 11 | 23 | 34 | 45 | 57 | 57 | 57 | 57 | 57 | 57 | 45 | 34 | 23 | 11 | 0 | 0 | 0 |
| 75% uptake | | | | | | | | | | | | | | | | | | |
| LAGB | NA | 1456 | 2911 | 4367 | 5822 | 7278 | 7278 | 7278 | 7278 | 7278 | 7278 | 5822 | 4367 | 2911 | 1456 | 0 | 0 | 0 |
| SG | NA | 188 | 376 | 565 | 753 | 941 | 941 | 941 | 941 | 941 | 941 | 753 | 565 | 376 | 188 | 0 | 0 | 0 |
| RYGB | NA | 24 | 49 | 73 | 97 | 122 | 122 | 122 | 122 | 122 | 122 | 97 | 73 | 49 | 24 | 0 | 0 | 0 |

_NA/previous year_

### Appendix 7.1.3 Treating New Eligible patients and a 5-year program to treat existing eligible patients (as of 2019-20)

| **uptake** | **2022-23** | **2023-24** | **2024-25** | **2025-26** | **2026-27** | **2027-28** | **2028-29** | **2029-30** | **2030-31** | **2031-32** | **2032-33** | **2033-34** | **2034-35** | **2035-36** | **2036-37** | **2037-38** | **2038-39** | **2039-40** |
| --- | --- | --- | --- | --- | --- | --- | --- | --- | --- | --- | --- | --- | --- | --- | --- | --- | --- | --- |
| 20% uptake | | | | | | | | | | | | | | | | | | |
| LAGB | NA | 444 | 889 | 1336 | 1785 | 2235 | 2299 | 2365 | 2433 | 2433 | 2433 | 1989 | 1543 | 1097 | 648 | 198 | 134 | 68 |
| SG | NA | 57 | 115 | 173 | 231 | 289 | 297 | 306 | 314 | 314 | 314 | 257 | 200 | 142 | 84 | 26 | 17 | 9 |
| RYGB | NA | 7 | 15 | 22 | 30 | 37 | 38 | 40 | 41 | 41 | 41 | 33 | 26 | 18 | 11 | 3 | 2 | 1 |
| 35% uptake | | | | | | | | | | | | | | | | | | |
| LAGB | NA | 777 | 1556 | 2338 | 3123 | 3911 | 4023 | 4138 | 4257 | 4257 | 4257 | 3480 | 2701 | 1919 | 1134 | 346 | 234 | 119 |
| SG | NA | 100 | 201 | 302 | 404 | 506 | 520 | 535 | 550 | 550 | 550 | 450 | 349 | 248 | 147 | 45 | 30 | 15 |
| RYGB | NA | 13 | 26 | 39 | 52 | 65 | 67 | 69 | 71 | 71 | 71 | 58 | 45 | 32 | 19 | 6 | 4 | 2 |
| 75% uptake | | | | | | | | | | | | | | | | | | |
| LAGB | NA | 1664 | 3334 | 5010 | 6692 | 8381 | 8621 | 8868 | 9122 | 9122 | 9122 | 7458 | 5788 | 4112 | 2430 | 741 | 501 | 254 |
| SG | NA | 215 | 431 | 648 | 865 | 1083 | 1115 | 1146 | 1179 | 1179 | 1179 | 964 | 748 | 532 | 314 | 96 | 65 | 33 |
| RYGB | NA | 28 | 56 | 84 | 112 | 140 | 144 | 148 | 153 | 153 | 153 | 125 | 97 | 69 | 41 | 12 | 8 | 4 |

NA/previous year

## Appendix 7.2 Total (private sector)

### Appendix 7.2.1 Newly eligible patients only

| **uptake** | **2022-23** | **2023-24** | **2024-25** | **2025-26** | **2026-27** | **2027-28** | **2028-29** | **2029-30** | **2030-31** | **2031-32** | **2032-33** | **2033-34** | **2034-35** | **2035-36** | **2036-37** | **2037-38** | **2038-39** | **2039-40** |
| --- | --- | --- | --- | --- | --- | --- | --- | --- | --- | --- | --- | --- | --- | --- | --- | --- | --- | --- |
| 20% uptake | | | | | | | | | | | | | | | | | | |
| LAGB | NA | 26 | 52 | 79 | 107 | 135 | 165 | 195 | 226 | 226 | 226 | 201 | 174 | 147 | 119 | 91 | 61 | 31 |
| SG | NA | 3 | 7 | 10 | 14 | 17 | 21 | 25 | 29 | 29 | 29 | 26 | 23 | 19 | 15 | 12 | 8 | 4 |
| RYGB | NA | 0 | 1 | 1 | 2 | 2 | 3 | 3 | 4 | 4 | 4 | 3 | 3 | 2 | 2 | 2 | 1 | 1 |
| 35% uptake | | | | | | | | | | | | | | | | | | |
| LAGB | NA | 45 | 91 | 138 | 187 | 237 | 288 | 341 | 396 | 396 | 396 | 351 | 305 | 258 | 209 | 159 | 108 | 55 |
| SG | NA | 6 | 12 | 18 | 24 | 31 | 37 | 44 | 51 | 51 | 51 | 45 | 39 | 33 | 27 | 21 | 14 | 7 |
| RYGB | NA | 1 | 2 | 2 | 3 | 4 | 5 | 6 | 7 | 7 | 7 | 6 | 5 | 4 | 3 | 3 | 2 | 1 |
| 75% uptake | | | | | | | | | | | | | | | | | | |
| LAGB | NA | 96 | 194 | 296 | 400 | 507 | 618 | 731 | 848 | 848 | 848 | 752 | 654 | 552 | 448 | 341 | 230 | 117 |
| SG | NA | 12 | 25 | 38 | 52 | 66 | 80 | 95 | 110 | 110 | 110 | 97 | 85 | 71 | 58 | 44 | 30 | 15 |
| RYGB | NA | 2 | 3 | 5 | 7 | 8 | 10 | 12 | 14 | 14 | 14 | 13 | 11 | 9 | 7 | 6 | 4 | 2 |

_NA/previous year_

### Appendix 7.2.2 Existing patients Only - assumes a 5-year program to treat all existing (2019-20) eligible patients

| **uptake** | **2022-23** | **2023-24** | **2024-25** | **2025-26** | **2026-27** | **2027-28** | **2028-29** | **2029-30** | **2030-31** | **2031-32** | **2032-33** | **2033-34** | **2034-35** | **2035-36** | **2036-37** | **2037-38** | **2038-39** | **2039-40** |
| --- | --- | --- | --- | --- | --- | --- | --- | --- | --- | --- | --- | --- | --- | --- | --- | --- | --- | --- |
| 20% uptake | | | | | | | | | | | | | | | | | | |
| LAGB | NA | 179 | 357 | 536 | 714 | 893 | 893 | 893 | 893 | 893 | 893 | 714 | 536 | 357 | 179 | 0 | 0 | 0 |
| SG | NA | 23 | 46 | 69 | 92 | 115 | 115 | 115 | 115 | 115 | 115 | 92 | 69 | 46 | 23 | 0 | 0 | 0 |
| RYGB | NA | 3 | 6 | 9 | 12 | 15 | 15 | 15 | 15 | 15 | 15 | 12 | 9 | 6 | 3 | 0 | 0 | 0 |
| 35% uptake | | | | | | | | | | | | | | | | | | |
| LAGB | NA | 312 | 625 | 937 | 1250 | 1562 | 1562 | 1562 | 1562 | 1562 | 1562 | 1250 | 937 | 625 | 312 | 0 | 0 | 0 |
| SG | NA | 40 | 81 | 121 | 162 | 202 | 202 | 202 | 202 | 202 | 202 | 162 | 121 | 81 | 40 | 0 | 0 | 0 |
| RYGB | NA | 5 | 10 | 16 | 21 | 26 | 26 | 26 | 26 | 26 | 26 | 21 | 16 | 10 | 5 | 0 | 0 | 0 |
| 75% uptake | | | | | | | | | | | | | | | | | | |
| LAGB | NA | 670 | 1339 | 2009 | 2678 | 3348 | 3348 | 3348 | 3348 | 3348 | 3348 | 2678 | 2009 | 1339 | 670 | 0 | 0 | 0 |
| SG | NA | 87 | 173 | 260 | 346 | 433 | 433 | 433 | 433 | 433 | 433 | 346 | 260 | 173 | 87 | 0 | 0 | 0 |
| RYGB | NA | 11 | 22 | 34 | 45 | 56 | 56 | 56 | 56 | 56 | 56 | 45 | 34 | 22 | 11 | 0 | 0 | 0 |

_NA/previous year_

### Appendix 7.2.3 Existing patients Only - assumes a 5-year program to treat all existing (2019-20) eligible patients

| **uptake** | **2022-23** | **2023-24** | **2024-25** | **2025-26** | **2026-27** | **2027-28** | **2028-29** | **2029-30** | **2030-31** | **2031-32** | **2032-33** | **2033-34** | **2034-35** | **2035-36** | **2036-37** | **2037-38** | **2038-39** | **2039-40** |
| --- | --- | --- | --- | --- | --- | --- | --- | --- | --- | --- | --- | --- | --- | --- | --- | --- | --- | --- |
| 20% uptake | | | | | | | | | | | | | | | | | | |
| LAGB | NA | 204 | 409 | 615 | 821 | 1028 | 1058 | 1088 | 1119 | 1119 | 1119 | 915 | 710 | 504 | 298 | 91 | 61 | 31 |
| SG | NA | 26 | 53 | 79 | 106 | 133 | 137 | 141 | 145 | 145 | 145 | 118 | 92 | 65 | 39 | 12 | 8 | 4 |
| RYGB | NA | 3 | 7 | 10 | 14 | 17 | 18 | 18 | 19 | 19 | 19 | 15 | 12 | 8 | 5 | 2 | 1 | 1 |
| 35% uptake | | | | | | | | | | | | | | | | | | |
| LAGB | NA | 357 | 716 | 1075 | 1437 | 1799 | 1851 | 1904 | 1958 | 1958 | 1958 | 1601 | 1242 | 883 | 522 | 159 | 108 | 55 |
| SG | NA | 46 | 93 | 139 | 186 | 233 | 239 | 246 | 253 | 253 | 253 | 207 | 161 | 114 | 67 | 21 | 14 | 7 |
| RYGB | NA | 6 | 12 | 18 | 24 | 30 | 31 | 32 | 33 | 33 | 33 | 27 | 21 | 15 | 9 | 3 | 2 | 1 |
| 75% uptake | | | | | | | | | | | | | | | | | | |
| LAGB | NA | 765 | 1534 | 2305 | 3078 | 3855 | 3966 | 4079 | 4196 | 4196 | 4196 | 3431 | 2662 | 1892 | 1118 | 341 | 230 | 117 |
| SG | NA | 99 | 198 | 298 | 398 | 498 | 513 | 527 | 542 | 542 | 542 | 443 | 344 | 245 | 144 | 44 | 30 | 15 |
| RYGB | NA | 13 | 26 | 39 | 52 | 65 | 66 | 68 | 70 | 70 | 70 | 57 | 45 | 32 | 19 | 6 | 4 | 2 |

_NA/previous year_

## Appendix 7.3 Total (public sector)

### Appendix 7.3.1 Newly eligible patients only

| **uptake** | **2022-23** | **2023-24** | **2024-25** | **2025-26** | **2026-27** | **2027-28** | **2028-29** | **2029-30** | **2030-31** | **2031-32** | **2032-33** | **2033-34** | **2034-35** | **2035-36** | **2036-37** | **2037-38** | **2038-39** | **2039-40** |
| --- | --- | --- | --- | --- | --- | --- | --- | --- | --- | --- | --- | --- | --- | --- | --- | --- | --- | --- |
| 20% uptake | | | | | | | | | | | | | | | | | | |
| LAGB | NA | 30 | 61 | 93 | 125 | 159 | 193 | 229 | 266 | 266 | 266 | 236 | 205 | 173 | 140 | 107 | 72 | 37 |
| SG | NA | 4 | 8 | 12 | 16 | 21 | 25 | 30 | 34 | 34 | 34 | 30 | 26 | 22 | 18 | 14 | 9 | 5 |
| RYGB | NA | 1 | 1 | 2 | 2 | 3 | 3 | 4 | 4 | 4 | 4 | 4 | 3 | 3 | 2 | 2 | 1 | 1 |
| 35% uptake | | | | | | | | | | | | | | | | | | |
| LAGB | NA | 53 | 107 | 162 | 219 | 278 | 338 | 401 | 465 | 465 | 465 | 412 | 358 | 303 | 245 | 187 | 126 | 64 |
| SG | NA | 7 | 14 | 21 | 28 | 36 | 44 | 52 | 60 | 60 | 60 | 53 | 46 | 39 | 32 | 24 | 16 | 8 |
| RYGB | NA | 1 | 2 | 3 | 4 | 5 | 6 | 7 | 8 | 8 | 8 | 7 | 6 | 5 | 4 | 3 | 2 | 1 |
| 75% uptake | | | | | | | | | | | | | | | | | | |
| LAGB | NA | 113 | 228 | 347 | 470 | 596 | 725 | 859 | 996 | 996 | 996 | 883 | 767 | 648 | 526 | 400 | 270 | 137 |
| SG | NA | 15 | 30 | 45 | 61 | 77 | 94 | 111 | 129 | 129 | 129 | 114 | 99 | 84 | 68 | 52 | 35 | 18 |
| RYGB | NA | 2 | 4 | 6 | 8 | 10 | 12 | 14 | 17 | 17 | 17 | 15 | 13 | 11 | 9 | 7 | 5 | 2 |

_NA/previous year_

### Appendix 7.3.2 Existing patients Only - assumes a 5-year program to treat all existing (2019-20) eligible patients

| **uptake** | **2022-23** | **2023-24** | **2024-25** | **2025-26** | **2026-27** | **2027-28** | **2028-29** | **2029-30** | **2030-31** | **2031-32** | **2032-33** | **2033-34** | **2034-35** | **2035-36** | **2036-37** | **2037-38** | **2038-39** | **2039-40** |
| --- | --- | --- | --- | --- | --- | --- | --- | --- | --- | --- | --- | --- | --- | --- | --- | --- | --- | --- |
| 20% uptake | | | | | | | | | | | | | | | | | | |
| LAGB | NA | 210 | 419 | 629 | 838 | 1048 | 1048 | 1048 | 1048 | 1048 | 1048 | 838 | 629 | 419 | 210 | 0 | 0 | 0 |
| SG | NA | 27 | 54 | 81 | 108 | 135 | 135 | 135 | 135 | 135 | 135 | 108 | 81 | 54 | 27 | 0 | 0 | 0 |
| RYGB | NA | 4 | 7 | 11 | 14 | 18 | 18 | 18 | 18 | 18 | 18 | 14 | 11 | 7 | 4 | 0 | 0 | 0 |
| 35% uptake | | | | | | | | | | | | | | | | | | |
| LAGB | NA | 367 | 734 | 1100 | 1467 | 1834 | 1834 | 1834 | 1834 | 1834 | 1834 | 1467 | 1100 | 734 | 367 | 0 | 0 | 0 |
| SG | NA | 47 | 95 | 142 | 190 | 237 | 237 | 237 | 237 | 237 | 237 | 190 | 142 | 95 | 47 | 0 | 0 | 0 |
| RYGB | NA | 6 | 12 | 18 | 25 | 31 | 31 | 31 | 31 | 31 | 31 | 25 | 18 | 12 | 6 | 0 | 0 | 0 |
| 75% uptake | | | | | | | | | | | | | | | | | | |
| LAGB | NA | 786 | 1572 | 2358 | 3144 | 3930 | 3930 | 3930 | 3930 | 3930 | 3930 | 3144 | 2358 | 1572 | 786 | 0 | 0 | 0 |
| SG | NA | 102 | 203 | 305 | 406 | 508 | 508 | 508 | 508 | 508 | 508 | 406 | 305 | 203 | 102 | 0 | 0 | 0 |
| RYGB | NA | 13 | 26 | 39 | 53 | 66 | 66 | 66 | 66 | 66 | 66 | 53 | 39 | 26 | 13 | 0 | 0 | 0 |

_NA Not Applicable (previous year)_

### Appendix 7.3.3 Existing patients Only - assumes a 5-year program to treat all existing (2019-20) eligible patients

| **uptake** | **2022-23** | **2023-24** | **2024-25** | **2025-26** | **2026-27** | **2027-28** | **2028-29** | **2029-30** | **2030-31** | **2031-32** | **2032-33** | **2033-34** | **2034-35** | **2035-36** | **2036-37** | **2037-38** | **2038-39** | **2039-40** |
| --- | --- | --- | --- | --- | --- | --- | --- | --- | --- | --- | --- | --- | --- | --- | --- | --- | --- | --- |
| 20% uptake | | | | | | | | | | | | | | | | | | |
| LAGB | NA | 240 | 480 | 721 | 964 | 1207 | 1241 | 1277 | 1314 | 1314 | 1314 | 1074 | 833 | 592 | 350 | 107 | 72 | 37 |
| SG | NA | 31 | 62 | 93 | 125 | 156 | 160 | 165 | 170 | 170 | 170 | 139 | 108 | 77 | 45 | 14 | 9 | 5 |
| RYGB | NA | 4 | 8 | 12 | 16 | 20 | 21 | 21 | 22 | 22 | 22 | 18 | 14 | 10 | 6 | 2 | 1 | 1 |
| 35% uptake | | | | | | | | | | | | | | | | | | |
| LAGB | NA | 419 | 840 | 1263 | 1686 | 2112 | 2173 | 2235 | 2299 | 2299 | 2299 | 1879 | 1459 | 1036 | 612 | 187 | 126 | 64 |
| SG | NA | 54 | 109 | 163 | 218 | 273 | 281 | 289 | 297 | 297 | 297 | 243 | 189 | 134 | 79 | 24 | 16 | 8 |
| RYGB | NA | 7 | 14 | 21 | 28 | 35 | 36 | 37 | 38 | 38 | 38 | 31 | 24 | 17 | 10 | 3 | 2 | 1 |
| 75% uptake | | | | | | | | | | | | | | | | | | |
| LAGB | NA | 899 | 1800 | 2705 | 3614 | 4526 | 4655 | 4789 | 4926 | 4926 | 4926 | 4027 | 3126 | 2220 | 1312 | 400 | 270 | 137 |
| SG | NA | 116 | 233 | 350 | 467 | 585 | 602 | 619 | 637 | 637 | 637 | 521 | 404 | 287 | 170 | 52 | 35 | 18 |
| RYGB | NA | 15 | 30 | 45 | 60 | 76 | 78 | 80 | 82 | 82 | 82 | 67 | 52 | 37 | 22 | 7 | 5 | 2 |

_NA Not Applicable (previous year)_

# Appendix 8: Costs

## Appendix 8.1 Total (Combined cost of private and public sector)

### Appendix 8.1.1 Newly eligible patients only

| **Uptake** | **2022-23** | **2023-24** | **2024-25** | **2025-26** | **2026-27** | **2027-28** | **2028-29** | **2029-30** |
| --- | --- | --- | --- | --- | --- | --- | --- | --- |
| Primary (P) surgery 20% uptake | | | | | | | | |
| LAGB | $13,729,459 | $14,121,267 | $14,524,791 | $14,940,393 | $15,368,452 | $15,809,354 | $16,263,502 | $16,731,308 |
| SG | $101,855,073 | $104,761,790 | $107,755,416 | $110,838,658 | $114,014,306 | $117,285,241 | $120,654,435 | $124,124,959 |
| RYGB | $5,172,395 | $5,320,004 | $5,472,026 | $5,628,599 | $5,789,864 | $5,955,968 | $6,127,063 | $6,303,302 |
| Total | $120,756,928 | $124,203,061 | $127,752,233 | $131,407,650 | $135,172,622 | $139,050,563 | $143,045,000 | $147,159,570 |
| Revisions (R) 20% uptake | | | | | | | | |
| LAGB | - | $696,246 | $1,412,362 | $2,148,941 | $2,906,597 | $3,685,959 | $4,487,681 | $5,312,434 |
| SG | - | $97,530 | $197,843 | $301,022 | $407,154 | $516,327 | $628,631 | $744,162 |
| RYGB | - | $14,122 | $28,647 | $43,587 | $58,955 | $74,763 | $91,024 | $107,753 |
| Total R |  | $807,898 | $1,638,852 | $2,493,551 | $3,372,705 | $4,277,049 | $5,207,337 | $6,164,348 |
| Total P + R | $120,756,928 | $125,010,959 | $129,391,085 | $133,901,201 | $138,545,327 | $143,327,612 | $148,252,336 | $153,323,918 |
| Primary surgery 35% uptake | | | | | | | | |
| LAGB | $24,026,554 | $24,712,218 | $25,418,383 | $26,145,688 | $26,894,791 | $27,666,370 | $28,461,128 | $29,279,789 |
| SG | $178,246,378 | $183,333,132 | $188,571,979 | $193,967,652 | $199,525,036 | $205,249,171 | $211,145,262 | $217,218,679 |
| RYGB | $9,051,691 | $9,310,006 | $9,576,045 | $9,850,048 | $10,132,262 | $10,422,945 | $10,722,359 | $11,030,779 |
| **Total P** | $211,324,624 | $217,355,356 | $223,566,407 | $229,963,388 | $236,552,088 | $243,338,486 | $250,328,749 | $257,529,247 |
| Revisions 35% uptake | | | | | | | | |
| LAGB |  | $1,218,431 | $2,471,634 | $3,760,647 | $5,086,544 | $6,450,429 | $7,853,442 | $9,296,759 |
| SG |  | $170,677 | $346,225 | $526,789 | $712,519 | $903,571 | $1,100,105 | $1,302,283 |
| RYGB |  | $24,714 | $50,133 | $76,278 | $103,171 | $130,835 | $159,293 | $188,568 |
| **Total R** |  | $1,413,822 | $2,867,991 | $4,363,714 | $5,902,235 | $7,484,835 | $9,112,839 | $10,787,610 |
| **Total P + R** | $211,324,624 | $218,769,178 | $226,434,398 | $234,327,102 | $242,454,323 | $250,823,321 | $259,441,589 | $268,316,856 |
| Primary surgery 75% uptake | | | | | | | | |
| LAGB | $51,485,472 | $52,954,753 | $54,467,965 | $56,026,475 | $57,631,694 | $59,285,079 | $60,988,132 | $62,742,404 |
| SG | $381,956,525 | $392,856,711 | $404,082,812 | $415,644,968 | $427,553,648 | $439,819,652 | $452,454,133 | $465,468,598 |
| RYGB | $19,396,482 | $19,950,014 | $20,520,097 | $21,107,245 | $21,711,990 | $22,334,882 | $22,976,485 | $23,637,384 |
| **Total P** | $452,838,479 | $465,761,477 | $479,070,873 | $492,778,688 | $506,897,332 | $521,439,613 | $536,418,749 | $551,848,386 |
| Revisions 75% uptake | | | | | | | | |
| LAGB | - | $2,610,924 | $5,296,358 | $8,058,530 | $10,899,737 | $13,822,347 | $16,828,804 | $19,921,626 |
| SG | - | $365,736 | $741,910 | $1,128,833 | $1,526,827 | $1,936,225 | $2,357,367 | $2,790,607 |
| RYGB | - | $52,958 | $107,427 | $163,453 | $221,081 | $280,361 | $341,341 | $404,074 |
| **Total R** |  | $3,029,618 | $6,145,695 | $9,350,816 | $12,647,646 | $16,038,933 | $19,527,513 | $23,116,307 |
| **Total P+R** | $452,838,479 | $468,791,096 | $485,216,568 | $502,129,504 | $519,544,978 | $537,478,546 | $555,946,261 | $574,964,692 |

### Appendix 8.1.2 Existing patients Only - assumes a 5-year program to treat all existing (2019-20) eligible patients

| **Uptake** | **2022-23** | **2023-24** | **2024-25** | **2025-26** | **2026-27** | **2027-28** | **2028-29** | **2029-30** |
| --- | --- | --- | --- | --- | --- | --- | --- | --- |
| Primary (P) surgery 20% uptake | | | | | | | | |
| LAGB | $95,902,221 | $95,902,221 | $95,902,221 | $95,902,221 | $95,902,221 | - | - | - |
| SG | $711,472,133 | $711,472,133 | $711,472,133 | $711,472,133 | $711,472,133 | - | - | - |
| RYGB | $36,129,913 | $36,129,913 | $36,129,913 | $36,129,913 | $36,129,913 | - | - | - |
| **Total P** | $843,504,267 | $843,504,267 | $843,504,267 | $843,504,267 | $843,504,267 | - | - | - |
| Revisions (R) 20% uptake | | | | | | | | |
| LAGB | - | $4,863,380 | $9,726,760 | $14,590,140 | $19,453,521 | $24,316,901 | $24,316,901 | $24,316,901 |
| SG | - | $681,259 | $1,362,518 | $2,043,777 | $2,725,035 | $3,406,294 | $3,406,294 | $3,406,294 |
| RYGB | - | $98,645 | $197,290 | $295,934 | $394,579 | $493,224 | $493,224 | $493,224 |
| **Total R** |  | $5,643,284 | $11,286,567 | $16,929,851 | $22,573,135 | $28,216,419 | $28,216,419 | $28,216,419 |
| **Total P + R** | $843,504,267 | $849,147,550 | $854,790,834 | $860,434,118 | $866,077,402 | $28,216,419 | $28,216,419 | $28,216,419 |
| Primary surgery 35% uptake | | | | | | | | |
| LAGB | $167,828,886 | $167,828,886 | $167,828,886 | $167,828,886 | $167,828,886 | - | - | - |
| SG | $1,245,076,233 | $1,245,076,233 | $1,245,076,233 | $1,245,076,233 | $1,245,076,233 | - | - | - |
| RYGB | $63,227,348 | $63,227,348 | $63,227,348 | $63,227,348 | $63,227,348 | - | - | - |
| **Total P** | $1,476,132,467 | $1,476,132,467 | $1,476,132,467 | $1,476,132,467 | $1,476,132,467 | - | - | - |
| Revisions 35% uptake | | | | | | | | |
| LAGB | - | $8,510,915 | $17,021,830 | $25,532,746 | $34,043,661 | $42,554,576 | $42,554,576 | $42,554,576 |
| SG | - | $1,192,203 | $2,384,406 | $3,576,609 | $4,768,812 | $5,961,015 | $5,961,015 | $5,961,015 |
| RYGB | - | $172,628 | $345,257 | $517,885 | $690,513 | $863,142 | $863,142 | $863,142 |
| **Total R** |  | $9,875,747 | $19,751,493 | $29,627,240 | $39,502,986 | $49,378,733 | $49,378,733 | $49,378,733 |
| **Total P + R** | $1,476,132,467 | $1,486,008,213 | $1,495,883,960 | $1,505,759,706 | $1,515,635,453 | $49,378,733 | $49,378,733 | $49,378,733 |
| Primary surgery 75% uptake | | | | | | | | |
| LAGB | $359,633,327 | $359,633,327 | $359,633,327 | $359,633,327 | $359,633,327 | - | - | - |
| SG | $2,668,020,499 | $2,668,020,499 | $2,668,020,499 | $2,668,020,499 | $2,668,020,499 | - | - | - |
| RYGB | $135,487,174 | $135,487,174 | $135,487,174 | $135,487,174 | $135,487,174 | - | - | - |
| **Total P** | $3,163,141,000 | $3,163,141,000 | $3,163,141,000 | $3,163,141,000 | $3,163,141,000 | - | - | - |
| Revisions 75% uptake | | | | | | | | |
| LAGB | - | $18,237,675 | $36,475,351 | $54,713,026 | $72,950,702 | $91,188,377 | $91,188,377 | $91,188,377 |
| SG | - | $2,554,721 | $5,109,441 | $7,664,162 | $10,218,883 | $12,773,603 | $12,773,603 | $12,773,603 |
| RYGB | - | $369,918 | $739,836 | $1,109,754 | $1,479,671 | $1,849,589 | $1,849,589 | $1,849,589 |
| **Total R** |  | $21,162,314 | $42,324,628 | $63,486,942 | $84,649,256 | $105,811,570 | $105,811,570 | $105,811,570 |
| **Total P + R** | $3,163,141,000 | $3,184,303,314 | $3,205,465,628 | $3,226,627,942 | $3,247,790,256 | $105,811,570 | $105,811,570 | $105,811,570 |

### Appendix 8.1.3 Treating New Eligible patients and a 5-year program to treat existing eligible patients (as of 2019-20)

| **Uptake** | **2022-23** | **2023-24** | **2024-25** | **2025-26** | **2026-27** | **2027-28** | **2028-29** | **2029-30** |
| --- | --- | --- | --- | --- | --- | --- | --- | --- |
| Primary surgery 20% uptake | | | | | | | | |
| LAGB | $109,631,680 | $110,023,488 | $110,427,011 | $110,842,614 | $111,270,672 | $15,809,354 | $16,263,502 | $16,731,308 |
| SG | $813,327,206 | $816,233,923 | $819,227,549 | $822,310,791 | $825,486,439 | $117,285,241 | $120,654,435 | $124,124,959 |
| RYGB | $41,302,308 | $41,449,917 | $41,601,939 | $41,758,512 | $41,919,777 | $5,955,968 | $6,127,063 | $6,303,302 |
| **Total P** | $964,261,195 | $967,707,327 | $971,256,499 | $974,911,917 | $978,676,889 | $139,050,563 | $143,045,000 | $147,159,570 |
| Revisions 20% uptake | | | | | | | | |
| LAGB | - | $5,559,627 | $11,139,122 | $16,739,082 | $22,360,117 | $28,002,860 | $28,804,582 | $29,629,334 |
| SG | - | $778,789 | $1,560,360 | $2,344,799 | $3,132,189 | $3,922,621 | $4,034,925 | $4,150,456 |
| RYGB | - | $112,767 | $225,937 | $339,522 | $453,534 | $567,987 | $584,248 | $600,977 |
| **Total R** |  | $6,451,182 | $12,925,420 | $19,423,402 | $25,945,840 | $32,493,467 | $33,423,755 | $34,380,767 |
| Total P + R | $964,261,195 | $974,158,509 | $984,181,919 | $994,335,319 | $1,004,622,729 | $171,544,031 | $176,468,755 | $181,540,337 |
| Primary surgery 35% uptake | | | | | | | | |
| LAGB | $191,855,440 | $192,541,104 | $193,247,269 | $193,974,574 | $194,723,676 | $27,666,370 | $28,461,128 | $29,279,789 |
| SG | $1,423,322,611 | $1,428,409,365 | $1,433,648,212 | $1,439,043,885 | $1,444,601,268 | $205,249,171 | $211,145,262 | $217,218,679 |
| RYGB | $72,279,039 | $72,537,354 | $72,803,393 | $73,077,396 | $73,359,610 | $10,422,945 | $10,722,359 | $11,030,779 |
| **Total P** | $1,687,457,090 | $1,693,487,823 | $1,699,698,874 | $1,706,095,854 | $1,712,684,555 | $243,338,486 | $250,328,749 | $257,529,247 |
| Revisions 35% uptake | | | | | | | | |
| LAGB | - | $9,729,346 | $19,493,464 | $29,293,393 | $39,130,205 | $49,005,005 | $50,408,018 | $51,851,335 |
| SG | - | $1,362,880 | $2,730,631 | $4,103,398 | $5,481,331 | $6,864,586 | $7,061,119 | $7,263,298 |
| RYGB | - | $197,342 | $395,389 | $594,163 | $793,685 | $993,977 | $1,022,434 | $1,051,709 |
| **Total R** |  | $11,289,568 | $22,619,484 | $33,990,954 | $45,405,221 | $56,863,568 | $58,491,572 | $60,166,342 |
| **Total P + R** | $1,687,457,090 | $1,704,777,391 | $1,722,318,358 | $1,740,086,808 | $1,758,089,776 | $300,202,054 | $308,820,321 | $317,695,589 |
| Primary surgery 75% uptake | | | | | | | | |
| LAGB | $411,118,800 | $412,588,080 | $414,101,292 | $415,659,802 | $417,265,021 | $59,285,079 | $60,988,132 | $62,742,404 |
| SG | $3,049,977,024 | $3,060,877,210 | $3,072,103,310 | $3,083,665,467 | $3,095,574,147 | $439,819,652 | $452,454,133 | $465,468,598 |
| RYGB | $154,883,656 | $155,437,188 | $156,007,271 | $156,594,419 | $157,199,165 | $22,334,882 | $22,976,485 | $23,637,384 |
| **Total P** | $3,615,979,479 | $3,628,902,478 | $3,642,211,873 | $3,655,919,688 | $3,670,038,332 | $521,439,613 | $536,418,749 | $551,848,386 |
| Revisions 75% uptake | | | | | | | | |
| LAGB | - | $20,848,600 | $41,771,709 | $62,771,557 | $83,850,439 | $105,010,725 | $108,017,182 | $111,110,003 |
| SG | - | $2,920,457 | $5,851,351 | $8,792,995 | $11,745,710 | $14,709,828 | $15,130,970 | $15,564,211 |
| RYGB | - | $422,876 | $847,263 | $1,273,206 | $1,700,753 | $2,129,950 | $2,190,931 | $2,253,663 |
| **Total R** |  | $24,191,932 | $48,470,323 | $72,837,758 | $97,296,902 | $121,850,503 | $125,339,083 | $128,927,877 |
| **Total P + R** | $3,615,979,479 | $3,653,094,410 | $3,690,682,196 | $3,728,757,446 | $3,767,335,234 | $643,290,116 | $661,757,831 | $680,776,262 |

## Appendix 8.2 Private sector

### Appendix 8.2.1 Newly eligible patients only

| **Uptake** | **2022-23** | **2023-24** | **2024-25** | **2025-26** | **2026-27** | **2027-28** | **2028-29** | **2029-30** |
| --- | --- | --- | --- | --- | --- | --- | --- | --- |
| Primary surgery 20% uptake | | | | | | | | |
| LAGB | $6,315,551 | $6,495,783 | $6,681,404 | $6,872,581 | $7,069,488 | $7,272,303 | $7,481,211 | $7,696,402 |
| SG | $46,853,334 | $48,190,423 | $49,567,492 | $50,985,783 | $52,446,581 | $53,951,211 | $55,501,040 | $57,097,481 |
| RYGB | $2,379,302 | $2,447,202 | $2,517,132 | $2,589,155 | $2,663,337 | $2,739,745 | $2,818,449 | $2,899,519 |
| **Total P** | $55,548,187 | $57,133,408 | $58,766,027 | $60,447,519 | $62,179,406 | $63,963,259 | $65,800,700 | $67,693,402 |
| Revisions 20% uptake | | | | | | | | |
| LAGB | - | $320,273 | $649,687 | $988,513 | $1,337,034 | $1,695,541 | $2,064,333 | $2,443,719 |
| SG | - | $44,864 | $91,008 | $138,470 | $187,291 | $237,510 | $289,170 | $342,314 |
| RYGB | - | $6,496 | $13,178 | $20,050 | $27,119 | $34,391 | $41,871 | $49,566 |
| **Total R** |  | $371,633 | $753,872 | $1,147,033 | $1,551,445 | $1,967,442 | $2,395,375 | $2,835,600 |
| **Total P + R** | $55,548,187 | $57,505,041 | $59,519,899 | $61,594,552 | $63,730,851 | $65,930,702 | $68,196,075 | $70,529,002 |
| Primary surgery 35% uptake | | | | | | | | |
| LAGB | $11,052,215 | $11,367,620 | $11,692,456 | $12,027,017 | $12,371,604 | $12,726,530 | $13,092,119 | $13,468,703 |
| SG | $81,993,334 | $84,333,241 | $86,743,110 | $89,225,120 | $91,781,516 | $94,414,619 | $97,126,820 | $99,920,592 |
| RYGB | $4,163,778 | $4,282,603 | $4,404,981 | $4,531,022 | $4,660,841 | $4,794,555 | $4,932,285 | $5,074,158 |
| **Total P** | $97,209,327 | $99,983,464 | $102,840,547 | $105,783,158 | $108,813,961 | $111,935,703 | $115,151,225 | $118,463,453 |
| Revisions 35% uptake | | | | | | | | |
| LAGB | - | $560,478 | $1,136,952 | $1,729,898 | $2,339,810 | $2,967,197 | $3,612,583 | $4,276,509 |
| SG | - | $78,511 | $159,263 | $242,323 | $327,759 | $415,643 | $506,048 | $599,050 |
| RYGB | - | $11,368 | $23,061 | $35,088 | $47,459 | $60,184 | $73,275 | $86,741 |
| **Total R** |  | $650,358 | $1,319,276 | $2,007,308 | $2,715,028 | $3,443,024 | $4,191,906 | $4,962,301 |
| **Total P + R** | $97,209,327 | $100,633,822 | $104,159,823 | $107,790,467 | $111,528,989 | $115,378,728 | $119,343,131 | $123,425,754 |
| Primary surgery 75% uptake | | | | | | | | |
| LAGB | $23,683,317 | $24,359,186 | $25,055,264 | $25,772,178 | $26,510,579 | $27,271,136 | $28,054,540 | $28,861,506 |
| SG | $175,700,002 | $180,714,087 | $185,878,093 | $191,196,685 | $196,674,678 | $202,317,040 | $208,128,901 | $214,115,555 |
| RYGB | $8,922,381 | $9,177,006 | $9,439,244 | $9,709,333 | $9,987,516 | $10,274,046 | $10,569,183 | $10,873,197 |
| **Total P** | $208,305,700 | $214,250,280 | $220,372,601 | $226,678,196 | $233,172,773 | $239,862,222 | $246,752,624 | $253,850,257 |
| Revisions 75% uptake | | | | | | | | |
| LAGB | - | $1,201,025 | $2,436,325 | $3,706,924 | $5,013,879 | $6,358,280 | $7,741,250 | $9,163,948 |
| SG | - | $168,239 | $341,279 | $519,263 | $702,341 | $890,663 | $1,084,389 | $1,283,679 |
| RYGB | - | $24,361 | $49,416 | $75,188 | $101,697 | $128,966 | $157,017 | $185,874 |
| **Total R** |  | $1,393,624 | $2,827,020 | $4,301,375 | $5,817,917 | $7,377,909 | $8,982,656 | $10,633,501 |
| **Total P + R** | $208,305,700 | $215,643,904 | $223,199,621 | $230,979,572 | $238,990,690 | $247,240,131 | $255,735,280 | $264,483,759 |

### Appendix 8.2.2 Existing patients Only - assumes a 5-year program to treat all existing (2019-20) eligible patients

| **Uptake** | **2022-23** | **2023-24** | **2024-25** | **2025-26** | **2026-27** | **2027-28** | **2028-29** | **2029-30** |
| --- | --- | --- | --- | --- | --- | --- | --- | --- |
| Primary surgery 20% uptake | | | | | | | | |
| LAGB | $44,115,021 | $44,115,021 | $44,115,021 | $44,115,021 | $44,115,021 | - | - | - |
| SG | $327,277,181 | $327,277,181 | $327,277,181 | $327,277,181 | $327,277,181 | - | - | - |
| RYGB | $16,619,760 | $16,619,760 | $16,619,760 | $16,619,760 | $16,619,760 | - | - | - |
| **Total P** | $388,011,963 | $388,011,963 | $388,011,963 | $388,011,963 | $388,011,963 | - | - | - |
| Revisions 20% uptake | | | | | | | | |
| LAGB | - | $2,237,155 | $4,474,310 | $6,711,465 | $8,948,619 | $11,185,774 | $11,185,774 | $11,185,774 |
| SG | - | $313,379 | $626,758 | $940,137 | $1,253,516 | $1,566,895 | $1,566,895 | $1,566,895 |
| RYGB | - | $45,377 | $90,753 | $136,130 | $181,506 | $226,883 | $226,883 | $226,883 |
| **Total R** |  | $2,595,911 | $5,191,821 | $7,787,732 | $10,383,642 | $12,979,553 | $12,979,553 | $12,979,553 |
| **Total P + R** | $388,011,963 | $390,607,873 | $393,203,784 | $395,799,694 | $398,395,605 | $12,979,553 | $12,979,553 | $12,979,553 |
| Primary surgery 35% uptake | | | | | | | | |
| LAGB | $77,201,288 | $77,201,288 | $77,201,288 | $77,201,288 | $77,201,288 | - | - | - |
| SG | $572,735,067 | $572,735,067 | $572,735,067 | $572,735,067 | $572,735,067 | - | - | - |
| RYGB | $29,084,580 | $29,084,580 | $29,084,580 | $29,084,580 | $29,084,580 | - | - | - |
| **Total P** | $679,020,935 | $679,020,935 | $679,020,935 | $679,020,935 | $679,020,935 | - | - | - |
| Revisions 35% uptake | | | | | | | | |
| LAGB | - | $3,915,021 | $7,830,042 | $11,745,063 | $15,660,084 | $19,575,105 | $19,575,105 | $19,575,105 |
| SG | - | $548,413 | $1,096,827 | $1,645,240 | $2,193,653 | $2,742,067 | $2,742,067 | $2,742,067 |
| RYGB | - | $79,409 | $158,818 | $238,227 | $317,636 | $397,045 | $397,045 | $397,045 |
| **Total R** |  | $4,542,843 | $9,085,687 | $13,628,530 | $18,171,374 | $22,714,217 | $22,714,217 | $22,714,217 |
| **Total P + R** | $679,020,935 | $683,563,778 | $688,106,622 | $692,649,465 | $697,192,308 | $22,714,217 | $22,714,217 | $22,714,217 |
| Primary surgery 75% uptake | | | | | | | | |
| LAGB | $165,431,330 | $165,431,330 | $165,431,330 | $165,431,330 | $165,431,330 | - | - | - |
| SG | $1,227,289,429 | $1,227,289,429 | $1,227,289,429 | $1,227,289,429 | $1,227,289,429 | - | - | - |
| RYGB | $62,324,100 | $62,324,100 | $62,324,100 | $62,324,100 | $62,324,100 | - | - | - |
| **Total P** | $1,455,044,860 | $1,455,044,860 | $1,455,044,860 | $1,455,044,860 | $1,455,044,860 | - | - | - |
| Revisions 75% uptake | | | | | | | | |
| LAGB | - | $8,389,331 | $16,778,661 | $25,167,992 | $33,557,323 | $41,946,654 | $41,946,654 | $41,946,654 |
| SG | - | $1,175,172 | $2,350,343 | $3,525,515 | $4,700,686 | $5,875,858 | $5,875,858 | $5,875,858 |
| RYGB | - | $170,162 | $340,324 | $510,487 | $680,649 | $850,811 | $850,811 | $850,811 |
| **Total R** |  | $9,734,664 | $19,469,329 | $29,203,993 | $38,938,658 | $48,673,322 | $48,673,322 | $48,673,322 |
| **Total P + R** | $1,455,044,860 | $1,464,779,525 | $1,474,514,189 | $1,484,248,853 | $1,493,983,518 | $48,673,322 | $48,673,322 | $48,673,322 |

### Appendix 8.2.3 Treating New Eligible patients and a 5-year program to treat existing eligible patients (as of 2019-20)

| **Uptake** | **2022-23** | **2023-24** | **2024-25** | **2025-26** | **2026-27** | **2027-28** | **2028-29** | **2029-30** |
| --- | --- | --- | --- | --- | --- | --- | --- | --- |
| Primary surgery 20% uptake | | | | | | | | |
| LAGB | $50,430,573 | $50,610,804 | $50,796,425 | $50,987,602 | $51,184,509 | $7,272,303 | $7,481,211 | $7,696,402 |
| SG | $374,130,515 | $375,467,604 | $376,844,673 | $378,262,964 | $379,723,762 | $53,951,211 | $55,501,040 | $57,097,481 |
| RYGB | $18,999,062 | $19,066,962 | $19,136,892 | $19,208,915 | $19,283,098 | $2,739,745 | $2,818,449 | $2,899,519 |
| **Total P** | $443,560,149 | $445,145,371 | $446,777,990 | $448,459,482 | $450,191,369 | $63,963,259 | $65,800,700 | $67,693,402 |
| Revisions 20% uptake | | | | | | | | |
| LAGB | - | $2,557,428 | $5,123,996 | $7,699,978 | $10,285,654 | $12,881,316 | $13,250,108 | $13,629,494 |
| SG | - | $358,243 | $717,766 | $1,078,607 | $1,440,807 | $1,804,406 | $1,856,066 | $1,909,210 |
| RYGB | - | $51,873 | $103,931 | $156,180 | $208,626 | $261,274 | $268,754 | $276,449 |
| **Total R** |  | $2,967,544 | $5,945,693 | $8,934,765 | $11,935,087 | $14,946,995 | $15,374,927 | $15,815,153 |
| Total P + R | $443,560,149 | $448,112,914 | $452,723,683 | $457,394,247 | $462,126,455 | $78,910,254 | $81,175,627 | $83,508,555 |
| Primary surgery 35% uptake | | | | | | | | |
| LAGB | $88,253,502 | $88,568,908 | $88,893,744 | $89,228,304 | $89,572,891 | $12,726,530 | $13,092,119 | $13,468,703 |
| SG | $654,728,401 | $657,068,308 | $659,478,177 | $661,960,187 | $664,516,583 | $94,414,619 | $97,126,820 | $99,920,592 |
| RYGB | $33,248,358 | $33,367,183 | $33,489,561 | $33,615,602 | $33,745,421 | $4,794,555 | $4,932,285 | $5,074,158 |
| **Total P** | $776,230,262 | $779,004,399 | $781,861,482 | $784,804,093 | $787,834,895 | $111,935,703 | $115,151,225 | $118,463,453 |
| Revisions 35% uptake | | | | | | | | |
| LAGB | - | $4,475,499 | $8,966,994 | $13,474,961 | $17,999,894 | $22,542,302 | $23,187,688 | $23,851,614 |
| SG | - | $626,925 | $1,256,090 | $1,887,563 | $2,521,412 | $3,157,710 | $3,248,115 | $3,341,117 |
| RYGB | - | $90,777 | $181,879 | $273,315 | $365,095 | $457,229 | $470,320 | $483,786 |
| **Total R** |  | $5,193,201 | $10,404,963 | $15,635,839 | $20,886,402 | $26,157,241 | $26,906,123 | $27,676,518 |
| **Total P + R** | $776,230,262 | $784,197,600 | $792,266,445 | $800,439,932 | $808,721,297 | $138,092,945 | $142,057,348 | $146,139,971 |
| Primary surgery 75% uptake | | | | | | | | |
| LAGB | $189,114,648 | $189,790,517 | $190,486,594 | $191,203,509 | $191,941,910 | $27,271,136 | $28,054,540 | $28,861,506 |
| SG | $1,402,989,431 | $1,408,003,516 | $1,413,167,523 | $1,418,486,115 | $1,423,964,107 | $202,317,040 | $208,128,901 | $214,115,555 |
| RYGB | $71,246,482 | $71,501,106 | $71,763,345 | $72,033,433 | $72,311,616 | $10,274,046 | $10,569,183 | $10,873,197 |
| **Total P** | $1,663,350,561 | $1,669,295,140 | $1,675,417,461 | $1,681,723,057 | $1,688,217,633 | $239,862,222 | $246,752,624 | $253,850,257 |
| Revisions 75% uptake | | | | | | | | |
| LAGB | - | $9,590,356 | $19,214,986 | $28,874,916 | $38,571,202 | $48,304,933 | $49,687,904 | $51,110,601 |
| SG | - | $1,343,410 | $2,691,622 | $4,044,778 | $5,403,027 | $6,766,521 | $6,960,246 | $7,159,537 |
| RYGB | - | $194,523 | $389,741 | $585,675 | $782,346 | $979,777 | $1,007,828 | $1,036,685 |
| **Total R** |  | $11,128,289 | $22,296,349 | $33,505,369 | $44,756,575 | $56,051,231 | $57,655,978 | $59,306,823 |
| **Total P + R** | $1,663,350,561 | $1,680,423,429 | $1,697,713,810 | $1,715,228,425 | $1,732,974,208 | $295,913,453 | $304,408,602 | $313,157,081 |

## Appendix 8.3 Public sector

### Appendix 8.3.1 Newly eligible patients only

| **Uptake** | **2022-23** | **2023-24** | **2024-25** | **2025-26** | **2026-27** | **2027-28** | **2028-29** | **2029-30** |
| --- | --- | --- | --- | --- | --- | --- | --- | --- |
| Primary surgery 20% uptake | | | | | | | | |
| LAGB | $7,413,908 | $7,625,484 | $7,843,387 | $8,067,812 | $8,298,964 | $8,537,051 | $8,782,291 | $9,034,906 |
| SG | $55,001,740 | $56,571,366 | $58,187,925 | $59,852,875 | $61,567,725 | $63,334,030 | $65,153,395 | $67,027,478 |
| RYGB | $2,793,093 | $2,872,802 | $2,954,894 | $3,039,443 | $3,126,527 | $3,216,223 | $3,308,614 | $3,403,783 |
| **Total P** | $65,208,741 | $67,069,653 | $68,986,206 | $70,960,131 | $72,993,216 | $75,087,304 | $77,244,300 | $79,466,168 |
| Revisions 20% uptake | | | | | | | | |
| LAGB | - | $375,973 | $762,676 | $1,160,428 | $1,569,562 | $1,990,418 | $2,423,348 | $2,868,714 |
| SG | - | $52,666 | $106,835 | $162,552 | $219,863 | $278,816 | $339,461 | $401,847 |
| RYGB | - | $7,626 | $15,469 | $23,537 | $31,836 | $40,372 | $49,153 | $58,187 |
| **Total R** |  | $436,265 | $884,980 | $1,346,517 | $1,821,261 | $2,309,606 | $2,811,962 | $3,328,748 |
| **Total P + R** | $65,208,741 | $67,505,918 | $69,871,186 | $72,306,649 | $74,814,477 | $77,396,911 | $80,056,262 | $82,794,916 |
| Primary surgery 35% uptake | | | | | | | | |
| LAGB | $12,974,339 | $13,344,598 | $13,725,927 | $14,118,672 | $14,523,187 | $14,939,840 | $15,369,009 | $15,811,086 |
| SG | $96,253,044 | $98,999,891 | $101,828,869 | $104,742,532 | $107,743,519 | $110,834,552 | $114,018,441 | $117,298,087 |
| RYGB | $4,887,913 | $5,027,403 | $5,171,064 | $5,319,026 | $5,471,422 | $5,628,390 | $5,790,074 | $5,956,621 |
| **Total P** | $114,115,297 | $117,371,892 | $120,725,860 | $124,180,229 | $127,738,128 | $131,402,782 | $135,177,525 | $139,065,793 |
| Revisions 35% uptake | | | | | | | | |
| LAGB | - | $657,953 | $1,334,682 | $2,030,750 | $2,746,734 | $3,483,232 | $4,240,859 | $5,020,250 |
| SG | - | $92,166 | $186,961 | $284,466 | $384,761 | $487,929 | $594,056 | $703,233 |
| RYGB | - | $13,345 | $27,072 | $41,190 | $55,712 | $70,651 | $86,018 | $101,827 |
| **Total R** |  | $763,464 | $1,548,715 | $2,356,406 | $3,187,207 | $4,041,811 | $4,920,933 | $5,825,309 |
| **Total P + R** | $114,115,297 | $118,135,356 | $122,274,575 | $126,536,635 | $130,925,334 | $135,444,594 | $140,098,458 | $144,891,102 |
| Primary surgery 75% uptake | | | | | | | | |
| LAGB | $27,802,155 | $28,595,567 | $29,412,701 | $30,254,296 | $31,121,115 | $32,013,942 | $32,933,591 | $33,880,898 |
| SG | $206,256,524 | $212,142,624 | $218,204,718 | $224,448,283 | $230,878,970 | $237,502,612 | $244,325,232 | $251,353,043 |
| RYGB | $10,474,100 | $10,773,007 | $11,080,852 | $11,397,912 | $11,724,475 | $12,060,836 | $12,407,302 | $12,764,187 |
| **Total P** | $244,532,779 | $251,511,198 | $258,698,271 | $266,100,492 | $273,724,559 | $281,577,391 | $289,666,124 | $297,998,128 |
| Revisions 75% uptake | | | | | | | | |
| LAGB | - | $1,409,899 | $2,860,033 | $4,351,606 | $5,885,858 | $7,464,068 | $9,087,554 | $10,757,678 |
| SG | - | $197,498 | $400,631 | $609,570 | $824,487 | $1,045,561 | $1,272,978 | $1,506,928 |
| RYGB | - | $28,597 | $58,011 | $88,264 | $119,384 | $151,395 | $184,324 | $218,200 |
| **Total R** |  | $1,635,994 | $3,318,675 | $5,049,441 | $6,829,729 | $8,661,024 | $10,544,857 | $12,482,806 |
| **Total P + R** | $244,532,779 | $253,147,192 | $262,016,947 | $271,149,932 | $280,554,288 | $290,238,415 | $300,210,981 | $310,480,934 |

### Appendix 8.3.2 Existing patients Only - assumes a 5-year program to treat all existing (2019-20) eligible patients

| **Uptake** | **2022-23** | **2023-24** | **2024-25** | **2025-26** | **2026-27** | **2027-28** | **2028-29** | **2029-30** |
| --- | --- | --- | --- | --- | --- | --- | --- | --- |
| Primary surgery 20% uptake | | | | | | | | |
| LAGB | $51,787,199 | $51,787,199 | $51,787,199 | $51,787,199 | $51,787,199 | - | - | - |
| SG | $384,194,952 | $384,194,952 | $384,194,952 | $384,194,952 | $384,194,952 | - | - | - |
| RYGB | $19,510,153 | $19,510,153 | $19,510,153 | $19,510,153 | $19,510,153 | - | - | - |
| **Total P** | $455,492,304 | $455,492,304 | $455,492,304 | $455,492,304 | $455,492,304 | - | - | - |
| Revisions 20% uptake | | | | | | | | |
| LAGB | - | $2,626,225 | $5,252,451 | $7,878,676 | $10,504,901 | $13,131,126 | $13,131,126 | $13,131,126 |
| SG | - | $367,880 | $735,760 | $1,103,639 | $1,471,519 | $1,839,399 | $1,839,399 | $1,839,399 |
| RYGB | - | $53,268 | $106,536 | $159,805 | $213,073 | $266,341 | $266,341 | $266,341 |
| **Total R** |  | $3,047,373 | $6,094,746 | $9,142,120 | $12,189,493 | $15,236,866 | $15,236,866 | $15,236,866 |
| **Total P + R** | $455,492,304 | $458,539,677 | $461,587,050 | $464,634,424 | $467,681,797 | $15,236,866 | $15,236,866 | $15,236,866 |
| Primary surgery 35% uptake | | | | | | | | |
| LAGB | $90,627,598 | $90,627,598 | $90,627,598 | $90,627,598 | $90,627,598 | - | - | - |
| SG | $672,341,166 | $672,341,166 | $672,341,166 | $672,341,166 | $672,341,166 | - | - | - |
| RYGB | $34,142,768 | $34,142,768 | $34,142,768 | $34,142,768 | $34,142,768 | - | - | - |
| **Total P** | $797,111,532 | $797,111,532 | $797,111,532 | $797,111,532 | $797,111,532 | - | - | - |
| Revisions 35% uptake | | | | | | | | |
| LAGB | - | $4,595,894 | $9,191,788 | $13,787,683 | $18,383,577 | $22,979,471 | $22,979,471 | $22,979,471 |
| SG | - | $643,790 | $1,287,579 | $1,931,369 | $2,575,158 | $3,218,948 | $3,218,948 | $3,218,948 |
| RYGB | - | $93,219 | $186,439 | $279,658 | $372,877 | $466,097 | $466,097 | $466,097 |
| **Total R** |  | $5,332,903 | $10,665,806 | $15,998,709 | $21,331,613 | $26,664,516 | $26,664,516 | $26,664,516 |
| **Total P + R** | $797,111,532 | $802,444,435 | $807,777,338 | $813,110,241 | $818,443,145 | $26,664,516 | $26,664,516 | $26,664,516 |
| Primary surgery 75% uptake | | | | | | | | |
| LAGB | $194,201,997 | $194,201,997 | $194,201,997 | $194,201,997 | $194,201,997 | - | - | - |
| SG | $1,440,731,069 | $1,440,731,069 | $1,440,731,069 | $1,440,731,069 | $1,440,731,069 | - | - | - |
| RYGB | $73,163,074 | $73,163,074 | $73,163,074 | $73,163,074 | $73,163,074 | - | - | - |
| **Total P** | $1,708,096,140 | $1,708,096,140 | $1,708,096,140 | $1,708,096,140 | $1,708,096,140 | - | - | - |
| Revisions 75% uptake | | | | | | | | |
| LAGB | - | $9,848,345 | $19,696,690 | $29,545,034 | $39,393,379 | $49,241,724 | $49,241,724 | $49,241,724 |
| SG | - | $1,379,549 | $2,759,098 | $4,138,647 | $5,518,197 | $6,897,746 | $6,897,746 | $6,897,746 |
| RYGB | - | $199,756 | $399,511 | $599,267 | $799,023 | $998,778 | $998,778 | $998,778 |
| **Total R** |  | $11,427,650 | $22,855,299 | $34,282,949 | $45,710,598 | $57,138,248 | $57,138,248 | $57,138,248 |
| **Total P + R** | $1,708,096,140 | $1,719,523,790 | $1,730,951,439 | $1,742,379,089 | $1,753,806,738 | $57,138,248 | $57,138,248 | $57,138,248 |

### Appendix 8.3.3 Treating New Eligible patients and a 5-year program to treat existing eligible patients (as of 2019-20)

| **Uptake** | **2022-23** | **2023-24** | **2024-25** | **2025-26** | **2026-27** | **2027-28** | **2028-29** | **2029-30** |
| --- | --- | --- | --- | --- | --- | --- | --- | --- |
| Primary surgery 20% uptake | | | | | | | | |
| LAGB | $59,201,107 | $59,412,683 | $59,630,586 | $59,855,011 | $60,086,163 | $8,537,051 | $8,782,291 | $9,034,906 |
| SG | $439,196,691 | $440,766,318 | $442,382,877 | $444,047,827 | $445,762,677 | $63,334,030 | $65,153,395 | $67,027,478 |
| RYGB | $22,303,246 | $22,382,955 | $22,465,047 | $22,549,596 | $22,636,680 | $3,216,223 | $3,308,614 | $3,403,783 |
| **Total P** | $520,701,045 | $522,561,957 | $524,478,510 | $526,452,435 | $528,485,520 | $75,087,304 | $77,244,300 | $79,466,168 |
| Revisions 20% uptake | | | | | | | | |
| LAGB | - | $3,002,198 | $6,015,126 | $9,039,104 | $12,074,463 | $15,121,544 | $15,554,474 | $15,999,840 |
| SG | - | $420,546 | $842,595 | $1,266,191 | $1,691,382 | $2,118,215 | $2,178,860 | $2,241,246 |
| RYGB | - | $60,894 | $122,006 | $183,342 | $244,908 | $306,713 | $315,494 | $324,527 |
| **Total R** |  | $3,483,638 | $6,979,727 | $10,488,637 | $14,010,754 | $17,546,472 | $18,048,828 | $18,565,614 |
| Total P + R | $520,701,045 | $526,045,595 | $531,458,236 | $536,941,072 | $542,496,274 | $92,633,777 | $95,293,128 | $98,031,782 |
| Primary surgery 35% uptake | | | | | | | | |
| LAGB | $103,601,937 | $103,972,196 | $104,353,525 | $104,746,270 | $105,150,785 | $14,939,840 | $15,369,009 | $15,811,086 |
| SG | $768,594,210 | $771,341,057 | $774,170,034 | $777,083,698 | $780,084,685 | $110,834,552 | $114,018,441 | $117,298,087 |
| RYGB | $39,030,681 | $39,170,171 | $39,313,832 | $39,461,794 | $39,614,190 | $5,628,390 | $5,790,074 | $5,956,621 |
| **Total P** | $911,226,829 | $914,483,424 | $917,837,392 | $921,291,761 | $924,849,660 | $131,402,782 | $135,177,525 | $139,065,793 |
| Revisions 35% uptake | | | | | | | | |
| LAGB | - | $5,253,847 | $10,526,471 | $15,818,432 | $21,130,311 | $26,462,703 | $27,220,330 | $27,999,721 |
| SG | - | $735,955 | $1,474,541 | $2,215,835 | $2,959,919 | $3,706,877 | $3,813,004 | $3,922,181 |
| RYGB | - | $106,565 | $213,510 | $320,848 | $428,590 | $536,747 | $552,115 | $567,923 |
| **Total R** |  | $6,096,367 | $12,214,521 | $18,355,115 | $24,518,819 | $30,706,327 | $31,585,449 | $32,489,825 |
| **Total P + R** | $911,226,829 | $920,579,791 | $930,051,913 | $939,646,876 | $949,368,479 | $162,109,109 | $166,762,974 | $171,555,618 |
| Primary surgery 75% uptake | | | | | | | | |
| LAGB | $222,004,152 | $222,797,563 | $223,614,697 | $224,456,293 | $225,323,111 | $32,013,942 | $32,933,591 | $33,880,898 |
| SG | $1,646,987,593 | $1,652,873,693 | $1,658,935,788 | $1,665,179,352 | $1,671,610,039 | $237,502,612 | $244,325,232 | $251,353,043 |
| RYGB | $83,637,174 | $83,936,082 | $84,243,926 | $84,560,986 | $84,887,549 | $12,060,836 | $12,407,302 | $12,764,187 |
| **Total P** | $1,952,628,919 | $1,959,607,338 | $1,966,794,411 | $1,974,196,632 | $1,981,820,699 | $281,577,391 | $289,666,124 | $297,998,128 |
| Revisions 75% uptake | | | | | | | | |
| LAGB | - | $11,258,244 | $22,556,723 | $33,896,641 | $45,279,237 | $56,705,791 | $58,329,278 | $59,999,402 |
| SG | - | $1,577,047 | $3,159,730 | $4,748,217 | $6,342,683 | $7,943,307 | $8,170,724 | $8,404,674 |
| RYGB | - | $228,353 | $457,522 | $687,531 | $918,406 | $1,150,173 | $1,183,103 | $1,216,978 |
| **Total R** |  | $13,063,643 | $26,173,975 | $39,332,389 | $52,540,327 | $65,799,272 | $67,683,105 | $69,621,053 |
| **Total P + R** | $1,952,628,919 | $1,972,670,981 | $1,992,968,386 | $2,013,529,021 | $2,034,361,026 | $347,376,662 | $357,349,229 | $367,619,182 |

# References

1. Australian Bureau of Statistics. National Health Survey: First results. 2018 [cited 2021 20 October]; Available from: <https://www.abs.gov.au/statistics/health/health-conditions-and-risks/national-health-survey-first-results/latest-release>.

2. Hayes A, Lung T, Bauman A,Howard K. Modelling obesity trends in Australia: unravelling the past and predicting the future. International journal of obesity, 2017. **41**(1): p. 178-185.

3. Sharman MJ, Breslin MC, Kuzminov A, Palmer AJ, Blizzard L, Hensher M,Venn AJ. Population estimates and characteristics of Australians potentially eligible for bariatric surgery: findings from the 2011–13 Australian Health Survey. Australian Health Review, 2017. **42**(4): p. 429-437.

4. ANZMOSS & Collaborative Public Bariatric Surgery Taskforce. Public Bariatric Surgery A National Framework. 2020: Australian & New Zealand Metabolic and Obesity Surgery Society.

5. Padwal RS, Pajewski NM, Allison DB,Sharma AM. Using the Edmonton obesity staging system to predict mortality in a population-representative cohort of people with overweight and obesity. Cmaj, 2011. **183**(14): p. E1059-E1066.

6. WebPlotDigitizer. WebPlotDigitizer. 2021 [cited 2021 04 April]; Available from: <https://automeris.io/WebPlotDigitizer/>.

7. Gómez JC, Lorido JA, Huelgas RG, Vidal MS, Tembra MS, Aguilar JV, Voces IM, Pérez EF, Rodríguez JF,Muñoz JE. Prevalence of obesity according to Edmonton staging in the Internal Medicine consultations. Results of the OBEMI study. Revista Clínica Española (English Edition), 2017. **217**(2): p. 71-78.

8. Lee PC, Ganguly S, Tan HC, Lim CH, Chan WH, Kovalik J-P, Eng A, Tan J, Lim E,Chua J. Attitudes and perceptions of the general public on obesity and its treatment options in Singapore. Obesity research & clinical practice, 2019. **13**(4): p. 404-407.

9. Sarwer DB, Ritter S, Wadden TA, Spitzer JC, Vetter ML,Moore RH. Attitudes about the safety and efficacy of bariatric surgery among patients with type 2 diabetes and a body mass index of 30–40 kg/m2. Surgery for Obesity and Related Diseases, 2013. **9**(5): p. 630-635.

10. Australian Institute of Health and Welfare. Australia's health 2020. 2020 [cited 2021 8 April]; Available from: <https://www.aihw.gov.au/reports-data/australias-health>.

11. Australian Institute of Health and Welfare. Admitted patient care 2018-19 6: what procedures were performed? 2020 [cited 2020 10 August].

12. Xia Q, Campbell JA, Ahmad H, de Graaff B, Si L, Otahal P, Ratcliffe K, Turtle J, Marrone J, Huque M, Hagan B, Green M,Palmer AJ. Resource utilization and disaggregated cost analysis of bariatric surgery in the Australian public healthcare system. The European Journal of Health Economics, 2021.

13. Morgan DJ, Platell C,Ho KM. The incidence and determinants of bariatric reoperations: a population-based cohort study. Surgery for Obesity and Related Diseases, 2020. **16**(5): p. 663-669.

14. Altieri MS, Yang J, Nie L, Blackstone R, Spaniolas K,Pryor A. Rate of revisions or conversion after bariatric surgery over 10 years in the state of New York. Surgery for Obesity and Related Diseases, 2018. **14**(4): p. 500-507.

15. Campbell JA, Hensher M, Davies D, Green M, Hagan B, Jordan I, Venn A, Kuzminov A, Neil A, Wilkinson S,Palmer AJ. Long-Term Inpatient Hospital Utilisation and Costs (2007-2008 to 2015-2016) for Publicly Waitlisted Bariatric Surgery Patients in an Australian Public Hospital System Based on Australia's Activity-Based Funding Model. Pharmacoecon Open, 2019. **3**(4): p. 599-618.

16. Independent Hospital Pricing Authority. National Hospital Cost Data Collection Report Public Sector, Round 23 (Financial Year 2018-19). 2021.

17. Australian Institute of Health and Welfare. Admitted patient care 2018-19: Australian hospital statistics [data set]. 2019 [cited 2020 05 June]; Available from: <https://www.aihw.gov.au/reports-data/myhospitals/sectors/admitted-patients>.

18. Gray AM, Clarke PM, Wolstenholme JL,Wordsworth S. Applied methods of cost-effectiveness analysis in healthcare. Vol. 3. 2011: Oxford University Press.
